# Resting-state EEG changes associated with cognitive decline in Parkinson’s disease: a systematic review

**DOI:** 10.1007/s00702-025-03065-0

**Published:** 2025-11-18

**Authors:** Milena Spoa, Sara Monti, Jovana Bjekić, Andrea Guerra, Eleonora Fiorenzato, Simone Cauzzo, Alessandra Bertoldo, Angelo Antonini

**Affiliations:** 1 Padova Neuroscience Center, University of Padova, Padua, Italy; 2 Human Neuroscience Group, Centre for Neuroscience and Neuromodulation, Institute for Medical Research, University of Belgrade, Belgrade, Serbia; 3 Department of Information Engineering, University of Padova, Padua, Italy; 4 Neurodegenerative Disease Unit, Centre for Rare Neurological Diseases (ERN-RND), Department of Neuroscience, Padua Neuroscience Center (PNC), University of Padova, Padua, Italy; 5 IRCCS San Camillo, Venice, Italy

**Keywords:** Electroencephalography, qEEG, Resting state, Cognitive decline, Parkinson’s disease

## Abstract

Cognitive decline is a common and disabling feature of Parkinson’s disease (PD), frequently progressing from mild cognitive impairment to Parkinson’s disease dementia. Due to substantial heterogeneity in PD phenotypes, current research is increasingly focused on identifying robust biomarkers to predict which individuals are at higher risk of cognitive deterioration relative to those likely to exhibit cognitive stability. Electroencephalography (EEG), a non-invasive and cost-efficient method for measuring neural activity, has shown potential in detecting early electrophysiological alterations associated with cognitive decline in PD. This review aims to summarise recent findings on the utility of resting-state quantitative EEG measures as candidate biomarkers for cognitive trajectory in PD. Relevant studies from over the last 10 years were retrieved through PubMed and Scopus, resulting in the inclusion and evaluation of 67 articles. Distinct EEG features, identified using various analytic approaches, were found to discriminate PD patients across different cognitive stages, as well as from those cognitively unimpaired and healthy controls. We suggest that EEG surrogate markers may be a valuable tool in conjunction with clinical and genetic variables to help early detection and monitoring, as well as development of personalised interventions for cognitive impairment in PD.

## Introduction

Parkinson’s disease (PD) is a progressive neurodegenerative disease estimated to affect 1–2% of adults over the age of 65, with a prevalence expected to double by 2030 (Ben-Shlomo et al. 2024). Cognitive deficits are common even early on in the disease course (Biundo et al. 2014; Schneider and Kortagere 2022), and a gradual broadening or worsening of the scope of cognitive impairment may pose challenges for therapeutic management (Van der Steen et al. 2019), exacerbate motor symptoms (Baiano et al. 2020) and significantly reduce the quality of life for both patients and caregivers (Antonini et al. 2012; Lawson et al. 2016). The clinical presentation of cognitive impairment in PD is highly heterogeneous (Gonzalez-Latapi et al. 2021), however, and predicting individual risk for progression to a more severe cognitive phenotype—including Parkinson’s disease dementia (PDD)—remains challenging (Puig-Davi et al. 2024), particularly when deficits are not yet clinically apparent.

Electroencephalography (EEG) provides a relatively accessible and cost-effective method for directly measuring neural activity and offers insights into various neurophysiological mechanisms (Sánchez-Dinorín et al. 2021). As such, it represents a valuable tool for investigating early or subtle neural changes associated with cognitive decline in PD (see Cozac et al. 2016b for an earlier review). With advancements in EEG analysis techniques facilitating the identification of novel biomarkers, the present review aims to systematically evaluate and update the current literature on the utility of resting-state EEG (rsEEG) as an indicator of cognitive dysfunction in PD. We first provide a brief overview of EEG and its main analytical approaches, and follow with a synthesis of the recent literature on EEG-derived neural markers of cognitive decline in PD.

### Overview of EEG

EEG recordings offer a direct, non-invasive measure of neural activity and, in contrast to imaging techniques, enable the evaluation of neural properties with high temporal resolution (Gavaret et al. 2023). In the absence of an exogenously imposed stimulus or task, EEG provides insight into the intrinsic oscillatory activity of the brain and its network dynamics (Mantini et al. 2007). Such resting state paradigms are typically recorded under brief eyes-open or -closed periods of cognitive disengagement (Perez et al. 2024), and are being increasingly employed to investigate possible neurophysiological change associated with pathology.

While conventional EEG enables a visual inspection of raw waveforms, relevant information for subsequent analyses or for comparisons with other types of data is typically extracted with quantitative EEG (qEEG); that is, the mathematical processing of raw EEG data using computational methods (Cozac et al. 2016b). Broadly speaking, and within the scope of this review, qEEG methods include spectral (frequency), source localisation, functional connectivity, microstate and machine learning based analyses.

#### Spectral analysis

Spectral analysis is a linear technique in which the time series of EEG data is decomposed into its frequency components using oscillatory basis functions, such as Fourier cosine basis functions or wavelets. Amplitudes of oscillations at each frequency bin are calculated and can be represented by their squares—known as *power*—such that the resulting *power spectrum* reflects the distribution of signal power over frequency (Cozac et al. 2016a, b). Power can be expressed either as absolute, corresponding to the total power within a given frequency band, or as relative to the total power summed across all frequency bands (Zhao et al. 2021). Frequency bands are often defined as in conventional EEG (i.e., delta (0.1–3 Hz), theta (4–7 Hz), alpha (8–12 Hz), beta (13–30 Hz), and gamma (31–90 Hz)), but may be further divided into sub-bands (i.e., low-alpha (8 – 10 Hz), high-alpha (10 – 12 Hz), low-beta (13–20 Hz), high-beta (21–30 Hz), low-gamma (31–40 Hz), mid-gamma (41–60 Hz), high-gamma (61–90 Hz)).

#### Source localisation analysis

Source localisation is rooted in solving both the *forward* and *inverse problem*; that is, which EEG scalp potential results from a given source of activity inside the brain, and which intracranial pattern of source activity generates a given EEG signal, respectively (Carboni et al. 2022). Because the propagation of activity generated by synchronised postsynaptic potentials from brain to scalp is not homogenous, the forward problem is typically addressed with biophysical head models that take into account the conductivity properties of different tissues as well as anatomic information of the head (Michel and He 2019). The inverse problem, on the other hand, can be solved only when a priori constraints about the neurophysiology of sources, electrical spread of activity and the distribution of neuronal activity are applied (Michel and He 2019). Minimum norm estimation (MNE; Hämäläinen and Ilmoniemi 1994), for instance, solves the inverse problem by finding the source configuration with minimum overall energy and is well-suited to model distributed cortical sources (Grech et al. 2008). Low resolution electrical tomography (LORETA; Pascual-Marqui et al. 1994) is a weighted minimum norm approach that applies spatial smoothing constraints to improve source localisation accuracy, especially for sources both near the surface and deeper in the brain (Grech et al. 2008).

#### Functional connectivity analysis

Functional connectivity (FC) refers to the statistical relationship between different neurophysiological regions (Friston 2011). It is commonly assessed by computing the correlation between signals recorded from different regions, and the strength of this correlation is used to determine the degree of synchronisation between such regions and infer how they are organised and what their function is (Cao et al. 2022; Khaleghi et al. 2024). Besides correlation, examples of undirected measures of FC derived in the time domain are cross-correlation analysis, mutual information, entropy and synchronisation likelihood (SL). Instead, typical measures derived from the frequency domain are coherence, Lagged Linear Connectivity (LLC), Phase Locking Value (PLV) and Phase Lag Index (PLI) (for a review of measures see Chiarion et al. 2023). An alternative approach to studying FC involves graph theory, in which the brain is represented as a network comprising nodes (representing brain regions) and edges (representing connections between them), allowing for the computation of both local and global properties (Hatlestad-Hall et al. 2023).

#### Microstate analysis

EEG microstates represent a dynamical view of how the spatial distribution of the electric potential on the scalp changes over time. Studies have shown that the brain's electrical field distribution at rest typically transitions between four distinct microstates, labelled A, B, C and D in clinical research (Yao et al. 2024). These microstates are defined as stable topographies of electrical potentials recorded across multiple electrodes, persisting for approximately 100 ms (Lehmann 1990). EEG microstates are highly reproducible, both within and across participants (Khanna et al. 2014), and while their precise functional meaning remains under debate, their topography, duration, occurrence, coverage, and sequence (microstate syntax) appear to systematically differ between health and disease (Michel and Koenig 2018; Milz et al. 2016).

#### Machine learning

Machine learning (ML) has become a powerful approach for identifying complex patterns in EEG data, improving signal interpretation and advancing applications in neuroscience and clinical diagnostics (Singh et al. 2022). For the purposes of this review, the objectives of ML will be summarised in terms of prediction, classification and clustering.

Prediction involves forecasting future outcomes based on current and past observations. Linear regression provides a first approach by modelling the relationship between EEG markers and cognitive outcomes, though more advanced techniques like Linear Mixed Models (LMMs) account for variations within and between subjects in longitudinal studies. Additionally, prediction is not limited to cognitive scores but extends to the signal itself. Techniques such as Linear Predictive Coding (O’Shaughnessy 1988) model EEG signals by estimating future values based on past observations, which can be useful in neurophysiological signal processing.

Classification, on the other hand, is used to categorise subjects based on predefined labels that are assigned, with a certain probability, after learning patterns from EEG-derived features. For instance, logistic regression maps input features to a probability score, making it useful for binary classification tasks, while more advanced methods, such as Support Vector Machines (SVM) (Cortes and Vapnik 1995), and ensemble models, like Random Forest (Breiman 2001), enhance accuracy by capturing complex patterns within the data.

Unlike classification and prediction, which rely on labelled data, clustering is an unsupervised learning approach that groups data based on intrinsic similarities, identifying naturally occurring subgroups. Algorithms such as k-Nearest Neighbours (kNN; Cover and Hart 1967) classify new data points based on their similarity to existing data, while deep learning models like Convolutional Neural Networks (CNN; Schirrmeister et al. 2017) can automatically extract complex hierarchical features, making them suitable for clustering high-dimensional data.

## Methods

The present review was conducted and reported following guidelines from the Preferred Reporting Items for Systematic reviews and Meta-Analyses (PRISMA; Page et al. 2021) framework.

### Inclusion and exclusion criteria

A systematic review of studies on rsEEG changes associated with cognitive impairment in PD was conducted, with articles obtained through a search of PubMed and Scopus. A search strategy was determined for PubMed pertaining to the three components of the research question (Table 1), and was then adjusted for the Scopus database. Only studies published between 2015 and 2025 were considered (see Cozac et al. 2016b for a synthesis of studies prior to 2015). Studies were included if they (a) evaluated cognitive status in PD patients, (b) employed resting-state (eyes-open and/or eyes-closed) EEG and (c) compared healthy controls and/or PD patients exhibiting normal cognition with PD patients exhibiting cognitive impairment on qEEG features OR evaluated qEEG features and cognitive performance in PD patients longitudinally OR correlated qEEG features of PD patients with performance on assessments of cognition. Exclusion criteria were: (a) animal studies, (b) review/perspective papers reporting no original data, (c) clinical trials, (d) case reports, (e) studies using other neuroimaging methods to collect resting-state data (e.g., magnetoencephalography (MEG), functional magnetic resonance imaging (fMRI)), (f) studies evaluating non-resting-state EEG, (g) studies evaluating EEG during sleep, (h) studies evaluating cognitive decline in relation to deep brain stimulation (DBS), (i) single-channel EEG studies and (j) studies published in languages other than English.

**Table 1:** Search strategy for PubMed database

1. parkinson disease [MeSH Terms]	10. dementia [Title/Abstract]
2. parkinson* [Title/Abstract]	11. PDD [Title/Abstract]
3. PD	12. MCI [Title/Abstract]
4. #1 OR #2 OR #3	13. “executive function*” [Title/Abstract]
5. electroencephalography [MeSH Terms]	14. attention [Title/Abstract]
6. electroencephalogra* [Title/Abstract]	15. memory [Title/Abstract]
7. eeg [Title/Abstract]	16. “processing speed” [Title/Abstract]
8. #5 OR #6 OR #7	17. visuospatial [Title/Abstract]
9. cognit*[Title/Abstract]	18. #9 OR #10 OR #11 OR #12 OR #13 OR #14 OR #15 OR #16 OR #17

### Data selection and extraction

Eligibility was determined independently by two authors (MS and SM). After duplicate removal, screening of title and abstract was performed to identify all potentially eligible records and screening of full texts to confirm inclusion. Discrepancies were resolved in a meeting between the two authors, and in case of a failed consensus, a third author (JB) was consulted. MS and SM then extracted the following data from each included paper: the first author, year of publication, design, sample nature and size, demographic characteristics (i.e., age, sex, education), clinical characteristics (i.e., disease duration, severity), performance on global cognitive tests, other possible neuropsychological assessments undertaken, cognitive subtyping criteria, EEG parameters and main outcomes from each analysis type. To ensure consistency and comparability across studies, group labels were standardised using common acronyms (i.e., HC for healthy controls, PD-NC for PD patients with normal cognition, PD-MCI for PD patients with mild cognitive impairment, and PDD for PD patients with dementia), regardless of the original terminology (e.g., CON for healthy controls). When necessary, participant groups were classified based on reported cognitive scores to align with these categories. When medication status was reported, this information was also included in the table (i.e., ‘ON’ or ‘OFF’ levodopa at time of recording). Similarly, alpha sub-bands were uniformly classified as *low alpha* (8–10 Hz) and *high alpha* (10–12 Hz); studies using alternative labels (e.g., alpha1, alpha2, alpha3) were reclassified according to reported frequency ranges. Given that qEEG methods are sensitive to the EEG system used and, in particular, to the number of electrodes, this information was also included in the tables.

## Results

### Search results

**Fig. 1:**
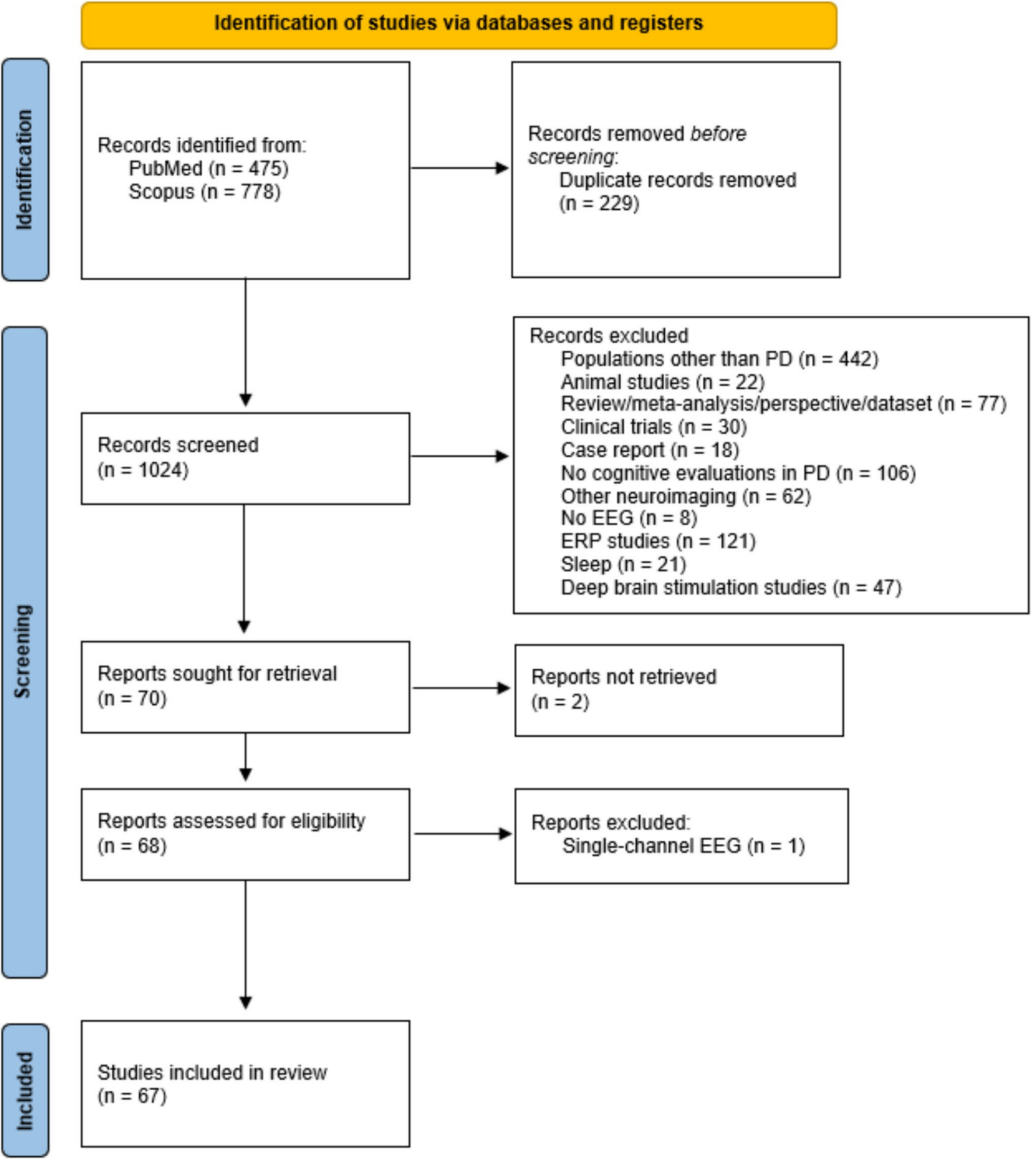
PRISMA flowchart of study selection procedure

The PRISMA flowchart describing the literature search and screening procedure with the number of articles remaining at each step is presented in Fig. 1. The literature search yielded a total of 1253 articles. After the removal of duplicates, 1024 articles remained. After the title and abstract screening process, 954 articles were excluded as a result of (a) population under study (i.e., not PD, animal studies), (b) design (i.e., reviews and other articles not reporting original data, clinical trials, case reports), and (c) methodological considerations (i.e., evaluations of EEG differences between PD patients and healthy controls not directly related to cognitive decline, other/no neuroimaging, non-resting-state EEG, EEG during sleep, evaluation of cognitive decline in relation to DBS). Two articles were removed because access to full text was restricted. After full-text screening, an additional article was excluded as a result of single-channel recordings. Sixty-seven studies were included in the review (see Table 2); those with sample sizes of PD patients below 30 were included in the tables but were not further elaborated on in text.

**Table 2:** Demographic and clinical characteristics of participants across studies

Study	Design	Sample	N	Age (years)	Sex (F/M)	Education (years)	Disease duration (years)	UPDRS-III score	MMSE/*MoCA score
				Mean (SD)/[min,max]		Mean (SD)/[min,max]	Mean (SD)/[min,max]	Mean (SD)/[min,max]	Mean (SD)/[min,max]
Abbood et al. (2024)	Cross-sectional	HC	36	69.77 (4.65)	12/24	9.22 (3.04)	–	–	–
		PDD	30	70.46 (3.81)	7/23	8.71 (2.12)	4.8 (1.1)	–	21.11 (2.90)
		DLB	36	71.12 (4.23)	10/26	9.00 (2.57)	4.5 (1.0)	–	20.32 (3.14)
Anjum et al. (2024)	Cross-sectional	HC	38	70.9 (7.6) all HC	23/26 all HC	16.5 (2.2) all HC			[22–30] all HC
		HC-MCI	11						
		PD-NC	47	68.5 (8.0) all PD	32/68 all PD	15.6 (3) all PD	–	12.4 (7.1) all PD, total score	[9–30] all PD
		PD-MCI	53				–		
		Out-of-sample dataset:							
		PD-CI	18	69.6 (7.8)	2/16	16.9 (3.2)	–	13.1 (8.2) all PD, total score	23.4 (1.1)
		PD-NC	14	65.0 (8.2)	9/5	16.1 (2.4)	–		27.6 (1.3)
Arnaldi et al. (2017)	Longitudinal 5y	Baseline:							
		Mainly motor PD (without MCI, RBD and OH)	19	67.26 (8.5)	9 M/10 F	12.68 (4.5)	–	12.1 (5.1)	29.4 (0.8) 0 MCI
		Intermediate PD (only MCI)	28	68.25 (6.8)	17 M/11 F	10.07 (4.0)	–	14.6 (6.7)	28.7 (0.9) 7 MCI
		Diffuse/malignant PD (with MCI and RBD/OH)	7	73.4 (4.4)	4 M/3 F	12.57 (4.1)	–	12.0 (3.4)	28.0 (1.9) 7 MCI
		Follow-up:							
		Cognitively Stable PD	33	66.42 (7.7)	19 M/14 F	11.70 (4.4)	–	12.5 (5.4)	28.9 (1.0) 8MCI
		Cognitively Worsened PD	21	71.95 (5.5)	11 M/10 F	10.71 (4.3)	–	14.8 (6.3)	28.8 (1.4) 6MCI
Babiloni et al. (2017)	Cross-sectional	HC	75	70.1 (0.8)	39/36	10.2 (0.5)	–	–	28.5 (0.1)
		PD-MCI	75	71.2 (0.8)	37/38	10.2 (0.6)	–	–	25.7 (0.3)
		AD-MCI	75	70.1 (0.7)	41/34	10.9 (0.5)	–	–	25.1 (0.3)
Babiloni et al. (2018a)	Cross-sectional	HC	40	72.9 (1.1)	24/16	8.5 (0.6)	–	–	28.7 (0.2)
		PDD	42	74.1 (1.1)	24/18	7.0 (0.6)	–	–	18.8 (0.7)
		ADD	42	73.3 (1.0)	25/17	8.1 (0.8)	–	–	18.9 (0.6)
		DLB	34	75.1 (1.1)	23/11	7.4 (0.8)	–	–	18.6 (0.8)
Babiloni et al. (2018b)	Cross-sectional	HC	75	70.1 (0.8)	39/36	10.2 (0.5)	–	–	28.5 (0.1)
		PD-MCI	75	71.2 (0.80)	37/38	10.2 (0.6)	–	–	25.7 (0.3)
		AD-MCI	75	70.1 (0.7)	41/34	10.9 (0.5)	–	–	25.1 (0.3)
Babiloni et al. (2019)	Cross-sectional	HC	50	70.8 (0.7)	26/24	9.2 (0.4)			28.4 (0.1)
		PD-NC	35	71.3 (1.2)	18/17	8.9 (0.7)	–	–	28.2 (0.3)
		PD-MCI	50	71.5 (1.0)	29/21	8.8 (0.6)	–	–	25.6 (0.4)
		PDD	35	73.8 (1.2)	21/14	7.9 (0.6)	–	–	19.0 (0.8)
Babiloni et al. (2020)	Cross-sectional	HC	65	70.7 (0.9)	32/33	8.6 (0.5)	–	–	28.5 (0.1)
		PD-NC	40	70.2 (1.1)	14/26	7.5 (0.6)	–	29.7 (2.2)	28.1 (0.2)
		PD-MCI	40	71.0 (1.0)	14/26	6.1 (0.6)	–	30.8 (2.1)	21.4 (0.5)
		AD	85	70.5 (0.7)	44/41	8.5 (0.5)	–	–	23.3 (0.4)
Babiloni et al. (2024)	Cross-sectional	HC	25	72.4 (1.6)	7/18	9.9 (0.8)	–	–	27.7 (0.3)
		PDD	73	72.7 (0.7)	12/61	9.4 (0.5)	–	–	18.9 (0.5)
		ADD	35	73.0 (1.1)	8/27	9.7 (0.5)	–	–	19.3 (0.8)
Betrouni et al. (2019)	Cross-sectional	PD-NC	28	60.5 (8.7)	8/20	13.6 (3.1)	7.7 (5.2)	26.0 (11.7)	–
		PD-with slight mental slowing	33	66.0 (6.6)	9/24	13.2 (4.1)	8.3 (7.4)	29.5 (12.2)	–
		PD-with mild cognitive deficits (especially EF)	43	67.0 (7.9)	16/27	11.6 (3.5)	8.8 (5.0)	28.7 (11.4)	–
		PD-with severe deficits in all cognitive domains (especially EF)	5	73.1 (5.2)	3/2	8.0 (1.4)	6.6 (3.5)	31.0 (12.7)	–
		PD-with severe deficits in all cognitive domains (especially WM and verbal episodic memory-recall)	9	67.5 (5.4)	0/9	10.5 (2.0)	12.0 (6.5)	29.0 (18.8)	–
Betrouni et al. (2022)	Cross-sectional	PD-NC	37	63.9 (7.4)	7/30	13.5 (3.9)	–	27.7 (12.5)	–
		PD-MCI (fronto-striatal subtype)	11	64.2 (10.0)	0/11	14.2 (3.5)	–	27.3 (11.2)	–
		PD-MCI (posterior-cortical subtype)	27	65.7 (8.2)	11/16	12.0 (3.0)	–	29.0 (11.5)	–
Bin Yoo et al. (2018)	Cross-sectional	HC	36	62.0 (9.0)	18/18	–	–	–	–
		PD	36	62.2 (9.1)	18/18	–	–	27.42 (10.22)	–
Burelo et al. (2024)	Cross-sectional	HC	41	75.81 (5.35)	14/27	–	–	–	29.17 (0.87)
		PDD	25	72.64 (4.34)	0/25	–	–	–	23.72 (2.4)
		AD	57	76.68 (7.44)	17/40	–	–	–	19.94 (4.37)
		DLB	44	75.25 (5.37)	4/40	–	–	–	23.1 (4.44)
Cai et al. (2021)	Cross-sectional	PD-NC	36	61.5 (9.9)	16/20	10.7 (3.3)	2	21.6 (11.0)	*26.5
		PD-MCI	32	66.6 (7.3)	15/17	6.2 (4.0)	3	24.7 (11.4)	*19
Carmona Arroyave et al. (2019)	Cross-sectional	PD-NC	22	61.8 (8.0)	8/14	11.5 (5.0)	4.4 (2.7)	28.3 (12.6)	26.5 (1.5) p < 0.001
		PD-MCI	14	66.1 (7.4)	4/10	11.9 (5.6)	6.2 (3.5)	34.6 (10.3)	20.9 (1.5)
		HC	36	63.3 (6.2)	12/24	12.4 (4.8)			26.6 (1.5)
Costa et al. (2023)	Cross-sectional	HC	10	53.2 (7.3)	6/4	–	–	–	–
		PD	10	58.5 (6.0)	4/6	–	6.3 (3.3)	–	–
Caviness et al. (2016a)	Cross-sectional	HC	51	86.0 (6.4)	–	–			
		ILBD	13	88.9 (6.0)	–	–	–	–	
		PD-NC	75	74.4 (9.2)	–	–	9.8 (5.6)	23.0 (13.8)	
		PD-MCI	28	76.7 (6.6)	–	–	12.2 (7.2)	28.3 (15.3)	
		PDD	31	78.9 (6.6)	–	–	14.7 (8.6)	38.9 (16.5)	
Caviness et al. (2016b)	Cross-sectional	PD	44	81.1 (6.8) at death	14/30	–	–	39 (16)	23.6 (4.8)
Chaturvedi et al. (2019)	Cross-sectional	PD-NC	43	67 [46,82]	17/26	–	4 [0,17]	15 [0,36]	*29 [27,30]
		PD-MCI	27	67 [53,84]	6/21	–	5 [0,23]	15 [0,41]	*29 [24,30]
Choi et al. (2024)	Cross-sectional	PD-NC	36	–	–	–	–	–	–
		PDD	37	–	–	–	–	–	–
		No brain pathology	48	–	–	–	–	–	–
		other	115	–	–	–	–	–	–
Chu et al. (2020)	Cross-sectional	HC	23	[60,74]	12/11	–	–	–	*28 [26,30]
		PD	23	[60,75]	15/8	–	3.2 (2.5)	18.4 [9,48]	*26.3 [19,30]
Chu et al. (2023a)	Cros-sectional	PD-NC	20	63.5 (6.2)	16/4	–	4.5 (4.4)	–	*27.7 (1.3)
		PD-MCI	13	62.0 (8.3)	7/6	–	3.3 (2.8)	–	*23.0 (1.8)
Chu et al. (2023b)	Cross-sectional	HC	22	63.8 (5.5)	11/11			–	–
		PD	29	62.4 (6.3)	20/9	–	–	15.8 (7.5)	*26.2 (2.9)
Conti et al. (2023)	Cross-sectional	HC	20	56.3 (8.8)	8/12	–			–
		PD	26	59.0 (9.4)	10/16	–	< 48 months	–	–
Conti et al. (2025)	Cross-sectional	HC	35	60.0 (6.8)	14/21	–	–	–	–
		PD pRBD+	28	65	8/20	–	1	25	*26.3
		PD pRBD−	35	62	13/22	–	0.5	22	*26.0
Cozac et al. (2016a, b)	Longitudinal 3y	PD	37	67	12/25	14	8	14	29
Eichelberger et al. (2017)	Cross-sectional	PD	57	67.21 (6.96)	17/40	14.67 (3.01)	5.25 (0.50)	14.77 (11.13)	28.70 (1.06)
Elkholy et al. (2023)	Cross-sectional	HC	47	62.85 (8.7)	26/21	–			
		PD	47	59.2 (8.3)	24/23	6.8 (6.1)	3.2 (2.5)	63.3 (27.5) total score	*20.6 (5.8)
Gimenez-Aparisi et al. (2023)	Cross-sectional	HC	20	68 (6.0)	12/8	–		5 (3.0)	28 (2.0)
		PD	13	68 (7.4)	7/6	–	5 (4.3)	29 (15.6)	28 (1.4)
Gschwandtner et al. (2023)	Longitudinal 3y	HC	72	[53,87]	35/37	[8,19]	–	–	[26,30]
		PD	75	[50,82]	25/50	[10,20]	[1,23]	–	[24,30]
Guner et al. (2017)	Cross-sectional	HC	39	65.9 (7.4)	19/20	6.0 (2.1)			28.5 (1.0)
		PD	45	66.5 (7.1)	21/24	5.7 (1.8)	1.8 (2.0)	7.6 (5.9)	28.0 (1.3)
Gu et al. (2016)	Longitudinal 2y	PD-MCI	17	61.7 [31.2–72.31]	5/12	10.4	–	–	27 (1.8)
		PDD	9	64.4 [45.3–75.9]	2/7	11.3	–	–	25 (1.1)
Hassan et al. (2017)	Cross-sectional	PD-NC	63	63.5 (7.9)	17/46	13.3 (3.6)	8.0 (6.4)	27.8 (11.9)	28.6 (1.4)
		PD-MCI	46	67.2 (7.7)	17/29	11.5 (3.5)	8.8 (4.9)	28.8 (11.0)	27 (2.1)
		PDD	15	70.0 (6.0)	3/12	9.4 (2.2)	10.6 (6.2)	32 (18.15)	24.1 (3.4)
Iyer et al. (2020)	Cross-sectional	HC	21	69.7 (5.9)	9/12	–	–	–	–
		PD	70	65.6 (8.8)	28/42	–	–	23.2 (8.6)	–
Jaramillo-Jimenez et al. (2021)	Cross-sectional	HC	36	63 (6)	12/24	12 (5)			27 (3)
		PD-NC	22						
		PD-MCI	14	63 (8)—all PD	12/24—all PD	12 (5)—all PD	5.2 (3.1)—all PD	28 (17)—all PD	25 (5)—all PD
Jennings et al. (2022)	Cross-sectional	HC	15	76.9 (4.5)	7/8	–	–	–	29.1 (0.8)
		PDD	17	73.8 (5.0)	2/15	–	1.4 (2.0)	–	23.1 (4.7)
		AD	12	74.4 (8.9)	8/4	–	1.9 (1.2)	–	20.0 (4.9)
		DLB	21	75.8 (6.7)	3/18	–	0.9 (0.6)	–	23.3 (4.3)
Jeong et al. (2015)	Cross-sectional	HC	26	71.5 (6.0)	15/11	–	–	–	–
		PDD	26	73.7 (6.9)	14/12	–	–	–	14.1 (4.3)
		AD	26	74.3 (6.7)	15/11	–	–	–	14.0 (4.4)
Keller et al. (2020)	Longitudinal 3y	HC	24	66.5	14	9/15			30
		PD-NC	42	66.5	14	18/24	2.5	14.5	29
Kozak et al. (2020)	Longitudinal 3y	PD-ND	47	66 [47, 80]	–	15 [9, 20]	6 [1, 24]	14 [2, 36]	–
Latreille et al. (2016)	Longitudinal 4.5y	HC	44	64.8 (10.9)	14/30	14.3 (3.7)			
		PD-ND	50	63.0 (8.5)	17/33	14.3 (4.1)	4.1 (3.1)	22.8 (10.1) ON	–
		PDD	18	70.2 (7.6)	5/13	14.4 (3.8)	5.7 (4.5)	21.5 (12.5) ON	–
Liu et al. (2023)	Cross-sectional	PD-NC	27	63.7 (5.7)	21/6	–	4.1 (3.9)	–	*27.8 (1.3)
		PD-MCI	13	62.0 (8.35)	7/6	–	3.3 (2.7)	–	*23.0 (1.8)
Liu et al. (2022)	Cross-sectional	HC	47	66.4 (9.3)	21/26	–			–
		PD-NC	47	62.4 (8.0)	26/21	–	4.1 (3.9)	22.1 (8.4)	27.4 (1.6)
		PDD	31	71.1 (7.0)	14/17	–	5.4 (3.5)	29.7 (11.3)	23.6 (2.4)
Lorek et al. (2023)	Cross-sectional	HC	11	63.4 (4.6)	6/5	–			–
		PD	11	65.4 (4.5)	5/6	–	–	26.1 (9.4)	–
Mano et al. (2022)	Cross-sectional	HC-MCI	10	70.4 (9.8)	4/6	4/6 (up to 19-year-old schooling/Further education and higher			26.4 (1.7)
		PD-MCI	20	72.7 (8.4)	8/12	8/12 (up to 18-year-old schooling/further education and higher)	–	–	26.4 (1.9)
Mehraram et al. (2020)	Cross-sectional	HC	18	76.2 (5.5)	7/11	–	–	1.2 (1.4) all	29.1 (0.8)
		PDD	21	73.3 (5.8)	1/20	–	–	24.5 (6.7) all	23.4 (3.4)
		AD	32	76.6 (7.7)	10/22	–	–	2.7 (3.1) all	20.1 (4.3)
		DLB	25	76.1 (6.2)	5/20	–	–	16.2 (7.5) all	22.6 (4.3)
Mostile et al. (2019)	Cross-sectional	PD-NC	56	65.4 (8.7)	27/29	8 (8)	1 (2)	UPDRS-ME (27.7 (11.6))	
		PD-MCI	46	66.1 (8.1)	21/25	5 (6.5)	2 (7)	30.5 (9.8)	
Novak et al. (2022)	Cross-sectional	HC	20	72.7 (8.4)	8/12	–			
		PD-NC	6			–	–		
		PDD	14	72.4 (8.1) whole PD group	08/12 whole PD group	–	–		
Orso et al. (2022)	Cross-sectional	PD	95	71.89 (7.53) [50,84]	35/60	10.92 (4.06)	–	20.72 (8.79)	28.09 (2.13)
Pal et al. (2020)	Cross-sectional	HC	26	57.6 (7.8)		14.1 (2.3)			29.3 (0.9)
		PD-NC	30	58.2 (7.5)		15.5 (2.6)	5.8 (2.7)	–	27.9 (1.6)
		PDD	28	62.0 (8.4)		13.3 (4.1)	6.4 (4.2)	–	22.0 (3.0)
Pal et al. (2021)	Cross-sectional	HC	20	57.4 (7.9)	–	–			29.9 (0.9)
		PD-NC	20	59.5 (7.9)	–	–	4	–	29.9 (0.9)
		PDD	18	61.1 (6.7)	–	–	4.5	–	22.1 (2.9)
Peláez Suárez et al. (2021)	Cross-sectional	HC	26	–	–	–			–
		PD-NC	15	58.4 (7.4)	1/4	–	7.7 (4.5)	31.7 (9.6)	–
		PD-MCI	15	62.7 (7.8)	1/4	–	8.0 (4.5)	36.0 (12.3)	–
Peraza et al. (2018)	Cross-sectional	HC	17	76.18 (5.65)	7/10	–	–	–	29.12 (0.86)
		PDD	21	73.29 (5.84)	2/19	–	1.41 (1.91)	25.57 (7.02)	22.71 (4.85)
		AD	26	76.46 (7.83)	8/18	–	1.77 (1.13)	2.28 (1.93)	20.08 (4.28)
		DLB	25	75.8 (76.5)	5/20	–	0.87 (0.63)	15.52 (8.2)	22.64 (4.3)
Puig-Davi et al. (2024)	Longitudinal 4y	Baseline:							
		HC	25	64.2 (5.9)	10/25	13.5 (4.9)	–	–	–
		PD	82	66.7 (8.3)	31/82	12.1 (4.7)	5.7 (3.1)	24.9 (7.7)	–
		Follow-up:							
		PD-stable	49	65.5 (8.9)	22/27	12.5 (4.4)	5.2 (2.7)	23.3 (7.4)	–
		PD-progressors	33	68.4 (6.9)	9/24	11.4 (5.0)	6.3 (3.7)	27.2 (7.8)	–
Rosenblum et al. (2023)	Cross-sectional	HC	22	73.4 (6.1) [61,84]	16/6	–	–	–	*26.5 (1.7)
		MCI	27	73.8 (5.0) [64,83]	13/14	–	–	–	*23.4 (3.4)
		PD	28	70.8 (5.6) [61,84]	7/21	–	3.5 (2.9)	19.2 (11.8)	*24.7 (2.9)
		DLB	21	71.7 (5.1) [62,81]	3/18	–	4.7 (2.1)	31.1 (13.6)	*18.6 (6.8)
Sánchez-Dinorín et al. (2021)	Cross-sectional	HC	15	61 (6)	10/5	14 (4)		2 (2)	27 (2)
		PD	30	63 (12)	12/18	12 (6)	6 (4)	26 (13)	26 (4)
Stylianou et al. (2018)	Cross-sectional	HC	21	76.19 (5.32)	7/14	–	–	–	29.19 (0.87)
		PDD	17	75.44 (4.66)	0/17	–	–	–	23.94 (2.59)
		AD	18	76.06 (7.81)	2/16	–	–	–	23.67 (1.68)
		DLB	17	75.71 (5.34)	2/15	–	–	–	25 (2.89)
Teramoto et al. (2016)	Cross-sectional	PD	68	68.0	33/35	–	5	–	–
Utianski et al. (2016)	Cross-sectional	HC	57	77	–	–			
		PD-NC	57	74.9 (8.2)	–	–	9.7 (5.6)	22 (14)	–
		PD-MCI	13	77.0 (5.2)	–	–	13.8 (7.9)	29 (12)	–
		PDD	18	80.6 (6.5)	–	–	17.0 (9.0)	43 (17)	–
Yassine et al. (2023)	Longitudinal 5y	G1	9	65.6 (6.4)	3/6	14.8 (2.9)	12.2 (5.8)	21.5 (12.7)	28.9 (1.7)/*27.7 (1.6)
		G2	22	72.6 (7.8)	7/15	14.9 (3.4)	9.3 (5.2)	15.9 (12.8)	28.6 (1.3)/*26.3 (1.9)
		G3	13	74.4 (6.7)	4/9	15.6 (2.8)	10.9 (3.5)	25.3 (13)	27.2 (2.6)/*21 (8)
Yi et al. (2022)	Cross-sectional	PD-NC	37	63.2 (5.6)	–	–	4.7 (5.3)	–	*27.6 (1.2)
		PD-MCI	30	60.7 (8.3)	–	–	3.6 (2.6)	–	*23.0 (1.5)
Yi et al. (2023)	Cross-sectional	HC	13	62.5 (5.0)	–	–			–
		PD-NC	20	63.5 (6.3)	–	–	4.5 (4.5)	–	*27.7 (1.3)
		PD-MCI	13	62.0 (8.3)	–	–	3.3 (2.7)	–	*23.0 (1.8)
Yılmaz et al. (2020)	Cross-sectional	HC	20	61.8 (7.6)	11/9	8.2 (4.4)			27.8 (1.5)
		PD-NC	19	60.2 (6.9)	7/12	9.5 (5.0)	4.3 (4.4)	14.5 (6.7)	26.3 (2.0)
		PD-MCI	18	67.7 (8.4)	4/14	4.7 (3.4)	5.5 (4.2)	17.1 (7.2)	22.5 (5.7)
		PDD	18	71.3 (8.0)	4/14	5.2 (5.2)	8.5 (5.1)	23.7 (11.7)	18.4 (4.6)
Zawiślak-Fornagiel et al. (2023a)	Cross-sectional	PD-NC	47	62.0 (12.1)	–	–	8.9 (6.9)	41.0 (15.1) (OFF)	–
		PD-MCI	33	68.7 (6.1)	–	–	8.3 (6.0)	43.4 (18.4) (OFF)	–
		PDD	28	71.9 (5.6)	–	–	13.5 (4.5)	59.8 (15.4) (OFF)	–
Zawiślak-Fornagiel et al. (2023b)	Cross-sectional	PD-NC	43	61.7 (12.4)	–	–	8.6 (6.8)	41.0 (14.8) (OFF)	–
		PD-MCI	30	68.2 (6.2)	–	–	8.4 (6.1)	44.0 (18.8) (OFF)	–
		PDD	25	71.3 (5.3)	–	–	13.8 (4.5)	58.4 (15.6) (OFF)	–
Zhang et al. (2021)	Cross-sectional	PD-NC	35	57.0 (11.9)	12/23	–	3.6 (3.6)	21.7 (9.4)	28.4 (1.2)/*25.9 (2.4)
		PD-MCI	36	61.1 (8.2)	18/18	–	2.8 (2.3)	21.9 (8.7)	26.5 (2.4)/*20.5 (4.4)
Zimmermann et al. (2015)	Cross-sectional	PD-NC	48	67.6(8.2)	16/32	14.6(3)	8.4(4)	14.8 (11.4) (ON)	–

*HC* healthy controls, *PD* Parkinson's disease, *PD-NC* Parkinson’s disease with normal cognition, *PD-MCI* Parkinson’s disease with mild cognitive impairment, *PDD* Parkinson’s disease with dementia, *PD-ND* Parkinson’s disease without dementia, *DLB* dementia with Lewy bodies, *ILBD* incidental Lewy body disease, *AD* Alzheimer's disease, *AD-MCI* Alzheimer’s disease with mild cognitive impairment, *ADD* Alzheimer’s disease with dementia, *RBD* rapid eye movement sleep behaviour disorder, *pRBD* premotor RBD, *OH* orthostatic hypotension, *UPDRS* Unified Parkinson’s Disease Rating Scale, *MoCA* Montreal Cognitive Assessment, *MMSE* Mini Mental State Evaluation, *EF* executive function, *WM* working memory. G1, G2 and G3 are clusters of electrophysiological profiles, defined by Yassine et al. (2023) as a mild-to-severe (diffuse) group with progressive cognitive decline (G3) and moderate (motor only) groups with more rapid motor progression (G1, G2)

### Spectral characteristics and cognitive manifestations

#### Global spectral characteristics

Fourteen studies evaluated global spectral features in relation to different cognitive states in PD (Table 3). A general shift toward slower oscillatory activity with cognitive worsening was reported in all studies. In particular, increases in global delta (Guner et al. 2017; Hassan et al. 2017; Yassine et al. 2023) and theta (Chaturvedi et al. 2019; Choi et al. 2024; Hassan et al. 2017; Yassine et al. 2023; Zawiślak-Fornagiel et al. 2023b) spectral power, as well as decreases in global alpha (Babiloni et al. 2017, 2019, 2020; Choi et al. 2024; Elkholy et al. 2023; Guner et al. 2017; Yassine et al. 2023) and beta (Chaturvedi et al. 2019; Elkholy et al. 2023; Hassan et al. 2017; Yassine et al. 2023) spectral power were observed in PD patients with cognitive impairment relative to those with normal cognition or controls.

**Table 3:** Summary of methodological choices and main spectral analysis findings

Study	EEG parameters	Main findings		
		Global spectral features	Regional spectral features	Associations with cognitive assessments
Abbood et al. (2024)	EO, 19 ch, 1 min, –	–	**δ (r)**: HC < PDD (F); HC < DLB (F, C); **θ (r)**: HC < DLB (F, C, T); PDD < DLB (F, C, T); **α (r)**: HC > PDD (F, C, T, P, O); HC > DLB (F, P, 0); PDD > DLB (F, C, T, P); **β (r)**: HC > PDD, DLB (F, C, T)	–
Anjum et al. (2024)	EO, 64 ch, 10 min, ON	–	–	Positive correlation between MoCA score and **β** (C-P), **α** (P), **γ** (C-P), **δ** (C-P) and **α/θ** ratio (P). Negative correlation between MoCA score and **θ** (F). Positive correlation between PVT score and **α** (P). Positive correlation between PVT, PCPST, PSMT, DCCST score and **β** (P). Negative correlation between PCPST, PSMT, DCCST score and **θ** (C). Positive correlation between FICAT score and **β** (P)
Babiloni et al. (2017)	EC, 19 ch 5 min, –	**mean TF**: HC > AD-MCI, PD-MCI; **mean IAF**: HC > AD-MCI > PD-MCI	See Table 4 for results	See Table 4 for results
Babiloni et al. (2018a)	EC, 19 ch, 5 min, –	**mean TF**: HC > ADD, PDD, DLB; **mean IAF**: HC > ADD, PDD, DLB	–	–
Babiloni et al. (2019)	–, 19 ch, –, –	**mean TF**: PDD < PD-MCI, PD, HC; PD-MCI < HC; **mean IAF**: PDD < PD-MCI, PD; PD-MCI, PD < HC	See Table 4 for results	–
Babiloni et al. (2020)	EC, 19 ch, 5 min, OFF/ON	**mean TF**: HC > AD > PD; HC > PD-MMSE−, PD-MMSE+; **mean IAF**: HC > AD > PD; HC > PD-MMSE−, PD-MMSE+; AD > PD-MMSE−, PD-MMSE+	–	–
Betrouni et al. (2019)	–, 128 ch, 10 min (extracted), ON	–	–	–
Betrouni et al. (2022)	EC, 128 ch, 10 min, –	**δ**: PD-FS > PD-NC; **θ**: PD-FS > PD-NC, PD-PC; **high-β**: PD-FS < PD-NC, PD-PC	**δ**: PD-FS > PD-NC (F, C); **θ**: PD-FS > PD-NC (F); **θ**: PD-FS > PD-NC, PD-PC (C, P); **high-β**: PD-FS < PD-NC (F); **high-β**: PD-FS < PD-NC, PD-PC (C, P)	–
Burelo et al. (2024)	EO/EC, 128 ch, 2 min, ON	–	Autoregressive: **EO θ (r)**: HC < PDD (C); HC < DLB (C, O, T); **EO α (r)**: no significant difference **EC θ (r)**: HC < PDD (F, C, T); HC < DLB (F, C, O, T); AD < PDD (F, C); AD < DLB (C). **EC α (r)**: HC < PDD (O); HC < DLB (O). Fast Fourier Transform: **EO θ (r)**: HC < PDD (F, C, O, T); HC < DLB (F, C, O, T). **EO α (r)**: no significant difference. **EC θ (r)**: HC < PDD (F, C, O, T); HC < DLB (F, C, O, T); AD < PDD (C, O, T); AD < DLB (F, C, O, T). **EC α (r)**: HC > PDD (C, O, T); HC > DLB (C, O, T); HC > AD (C, O, T)	–
Caviness et al. (2016a)	EC, 20 ch, –, ON	**δ (r)**: PDD(AD) > PDD(-D)	–	–
Chaturvedi et al. (2019)	EC, 256 ch, 12 min, –	**θ**: PD-MCI > PD-nMCI; **β**: PD-MCI < PD-nMCI	–	–
Choi et al. (2024)	EO/EC, –, 1 min, –	**θ (r)**: PD-MCI, ADPD-MCI > AD-MCI, MCI (NOS); **θ:α**: PD-MCI, ADPD-MCI > AD-MCI, MCI (NOS); **α (r)**: PD-MCI, ADPD-MCI < AD-MCI, MCI (NOS)	–	–
Cozac et al. (2016a, b)	EC, 256 ch, –, –	–	–	–
Eichelberger et al. (2017)	EC, 256 ch, 15 min, –	–	–	Increase in the **α/θ** ratio (P, O) predictive of increase in ROCF score
Elkholy et al. (2023)	EC, 19 ch, 3–4 min (extracted), –	**α, β**: PD-MCI < PD-NC	**δ, θ, α, β**: PD > HC (r-T); **δ, θ, α, β**: PD-MCI < PD-NC (b-F); **θ, α, β**: PD-MCI < PD-NC (b-T); **δ**: PD-MCI < PD-NC (l–T); **α, β**: PD-MCI < PD-NC (b-O); **θ**: PD-MCI < PD-NC (r-O)	–
Guner et al. (2017)	EC, 21 ch, 20 min, –	**δ (r), θ (r)**: PD > HC; **α (r)**: PD < HC	**δ (a)**, **θ (a)**: PD > HC (F, C, T, O, P); **β (a)**: PD > HC (F, P, O); **α (a)**: PD < HC (F, C, T, O, P); **[α + β]/[δ + θ]**: PD < HC (F, C, T, O, P)	Positive correlation between MMSE score and **[α + β]/[δ + θ]** (F, C, T, P, O)
Hassan et al. (2017)	EC, 128 ch, 10 min, –	**δ (r), θ (r)**: PDD > PD-MCI, PD-NC; **β (r)**: PDD < PD-MCI, PD-NC	–	
Jaramillo-Jimenez et al. (2021)	EC, 58 ch, 5 min, –	–	–	Positive correlation between JLO score and **α/θ** ratio (P, O)
Latreille et al. (2016)	EC, 12 ch, 10 min, –	–	**δ (a)**: PDD > PD-ND > HC (F, C, T, O, P); **α (peak)**: PDD < PD-ND, HC (O); **slow-to-fast ratio**: PDD > PD-ND, HC (F, C, T, O, P)	–
Liu et al. (2022)	EC, 19 ch, 15 min, –	**θ, α, β (a)**: no significant differences between PDD, PD-NC and HC	**δ**: PDD > PD-NC, HC (F, C, T, O, P); **θ**: PDD > PD-NC, HC (F, C, T); **α (peak)**: PDD < PD-NC, HC (O)	Negative correlation between MMSE score and absolute **δ** (F)
Mostile et al. (2019)	EC, 19 ch, –, –	–	**δ**: PD-MCI > PD-NC (l–T); **α**: PD-MCI < PD-NC (O)	–
Orso et al. (2022)	EC, 19 ch, 1 min, OFF	–	–	Positive correlation between α-θ ratio (P) and memory performance. Positive correlation between **α/θ** ratio (P) and visuospatial performance
Pal et al. (2020)	EC, 128 ch, 3 min, –	–	–	–
Peláez Suárez et al. (2021)	–, –, 30 min, ON	–	–	–
Puig-Davi et al. (2024)	EC, 19 ch, 8 min, –	–	**δ**: PD-prog > PD-stab (T); **θ**: PD-prog > PD-stab (F, T, O, P)	–
Stylianou et al. (2018)	EC, 128 ch, –, ON	–	**θ (r)**: PDD, DLB > AD, HC (F, C, T, O); **α (r)**: PDD < HC (F, C, T, O), PD < AD (T, O), DLB < HC (F, O, T), DLB < AD (F, O, T); **β (r)**: PDD < HC (F, C, O, T), PDD < AD (F, C, O), DLB < AD, HC (F, C, O, T)	No significant correlations found for PDD alone
Yassine et al. (2023)	EC, 256 ch, 12 min, –	At baseline. **δ (r), θ (r)**: G3 > G1, G2; **α (r), β (r)**: G3 < G1, G2. **SMN δ**: G1 < G3; **SMN β**: G1 > G3; **DMN low-α**: G2 > G3; **FTN high-α**: G1, G2 > G3. At 5 year follow-up. G1, G2: relatively constant power spectrum; G3: increase in **δ**, **θ** (r)	–	Positive correlation between MoCA score and DMN **low-α** power at 3 year follow-up
Yi et al. (2023)	EC, 19 ch, 15 min, –	–	–	–
Yılmaz et al. (2020)	EO/EC, 32 ch, –, ON	–	–	Positive correlation between MMSE score and **α** (P, T, O)
Zawiślak-Fornagiel et al. (2023b)	EC, 19 ch, 20 min, ON	**θ (a)**: PDD > PD-NC	**β (r)**: PDD < PD-NC (T, P, O); **θ (r)**: PDD > PD-NC (l–T, b-O); **α-θ ratio**: PDD < PD-NC (r-F, b-C, b-T, b-P, b-O); **spectral-power ratio**: PDD < PD-NC (b-T, b-O)	–
Zhang et al. (2021)	EO, 16 ch, 30 min, –	–	**δ**: PD-MCI > PD-NC (l-P, r-O); **θ**: PD-MCI > PD-NC (l-C, l-P, l–T)	–
Zimmermann et al. (2015)	EC, 256 ch, 15 min, –	–	–	Increase in EEG median frequency predictive of better performance on tests of attention, executive function, fluency, episodic long-term memory

If studies reported the number of electrodes rather than the number of channels, a one-to-one correspondence was assumed and reported in the table unless otherwise specified

*EC* eyes closed, *EO* eyes open, *ch* channels, *HC* healthy controls, *PD* Parkinson’s disease, *PD-NC* Parkinson’s disease with normal cognition, *PD-MCI* Parkinson’s disease with mild cognitive impairment, *PDD* Parkinson’s disease with dementia, *PD-ND* Parkinson’s disease without dementia, *PD-MMSE+/−* Parkinson’s disease with low/high MMSE score (both groups include both PD-MCI and PD-NC) defined by Babiloni et al. (2020), *DLB* dementia with Lewy bodies, *AD* Alzheimer’s disease, *AD-MCI* Alzheimer’s disease with mild cognitive impairment, *ADD* Alzheimer’s disease with dementia, *r* relative, *a* absolute, *F* frontal, *T* temporal, *P* parietal, *C* central, *O* occipital, *L* limbic, *FTN* fronto-temporal network, *DMN* default mode network, *SMN* sensorimotor network, *MoCA* Montreal Cognitive Assessment, *MMSE* Mini Mental State Evaluation, *PVT* Picture Vocabulary test, *PCPST* Pattern Comparison Processing Speed test, *PSMT* Picture Sequence Memory test, *DCCST* Dimensional Change Card Sorting test, *FICAT* Flanker Inhibitory Control and Attention test, *ROCF* Rey–Osterrieth Complex Figure test, *JLO* Judgement of Line Orientation test. G1, G2 and G3 are clusters of electrophysiological profiles, defined by Yassine and colleagues (2023) as a mild-to-severe (diffuse) group with progressive cognitive decline (G3) and moderate (motor only) groups with more rapid motor progression (G1, G2)

The frequency at which dominant brain activity shifted from the theta to the alpha band—or the *mean transition frequency*—was lower in PDD patients compared to healthy controls (HC) (Babiloni et al. 2018a), PD patients with mild cognitive impairment (PD-MCI) and PD patients with normal cognition (PD-NC) (Babiloni et al. 2019), as well as in PD-MCI compared to HC (Babiloni et al. 2017, 2020). The individual alpha frequency, defined as the maximum power density peak between 6 and 14 Hz, was similarly lower in PDD compared to PD-MCI (Babiloni et al. 2019) and HC (Babiloni et al. 2018a), and in PD-MCI compared to HC (Babiloni et al. 2017, 2019, 2020). Similar results were reported by Yassine et al. (2023), who characterised PD subtypes based on distinct electrophysiological profiles and investigated neurophysiological progression longitudinally in terms of clinical status. In particular, a mild-to-severe subtype of PD with progressive cognitive decline (i.e., G3) was characterised by higher relative delta and theta power, and lower relative alpha and beta power compared to the moderate, primarily-motor subtypes (i.e., G1, G2) at baseline. At a 5-year follow-up, G3 patients showed increases in slow-frequency bands as well as poor global cognition and executive function performance, while G1 and G2 patients maintained a relatively constant power spectrum and cognitive status (Yassine et al. 2023).

One study evaluating a fronto-striatal subtype (PD-FS) and a posterior-cortical subtype (PD-PC) of MCI in PD reported an increase in global delta and theta spectral power and a decrease in high-beta spectral power in PD-FS patients compared to PD-PC patients and PD-NC (Betrouni et al. 2022). Another study comparing EEG features in autopsy-confirmed PD and Alzheimer’s disease (AD) cases exhibiting MCI reported increased global theta band activity and decreased global alpha band activity in both PD-MCI and ADPD-MCI patients relative to AD-MCI patients (Choi et al. 2024). Higher global relative delta band power activity was reported in PDD patients with AD in comparison to PDD patients without AD (Caviness et al. 2016a).

#### Topographical distribution of spectral power

Twelve studies addressed regional, or local, spectral power in relation to cognitive state in PD. The most consistent finding was that of decreased *alpha* activity in the occipital regions of cognitively impaired PD patients compared to PD-NC or HC (Abbood et al. 2024; Babiloni et al. 2019; Elkholy et al. 2023; Latreille et al. 2016; Liu et al. 2022; Mostile et al. 2019). Decreased alpha band activity was also reported in frontal and temporal regions (Abbood et al. 2024; Elkholy et al. 2023) as well as in central and parietal regions (Abbood et al. 2024) in PD patients with cognitive impairment compared to PD-NC and HC.

*Delta* band activity was generally higher in frontal and central regions (Abbood et al. 2024; Babiloni et al. 2019; Latreille et al. 2016; Liu et al. 2022), as well as in temporal (Guner et al. 2017; Latreille et al. 2016; Liu et al. 2022; Mostile et al. 2019), parietal (Guner et al. 2017; Latreille et al. 2016; Liu et al. 2022; Zhang et al. 2021), and occipital (Guner et al. 2017; Latreille et al. 2016; Liu et al. 2022; Zhang et al. 2021) regions in PD patients with cognitive impairment compared to PD-NC or HC. Moreover, PD patients who developed more severe cognitive impairment had higher baseline delta band activity in temporal regions, as well as higher frontal, temporal and occipital theta band activity compared to those whose cognitive status remained relatively stable at a 4-year follow up (Puig-Davi et al. 2024). In one study, however, relative power in both theta and delta bands was reportedly lower in PD patients with cognitive impairment compared to PD-NC in temporal and frontal regions (Elkholy et al. 2023). Moreover, one study comparing MCI subtypes of PD reported that increased delta band activity in frontal and central regions was observed only in patients with fronto-striatal mild cognitive deficits, and not in those with posterior-cortical deficits or PD-NC (Betrouni et al. 2022).

*Theta* band activity was generally higher in temporal regions (Liu et al. 2022; Zawiślak-Fornagiel et al. 2023b; Zhang et al. 2021), as well as in frontal (Liu et al. 2022), parietal (Zhang et al. 2021), central (Liu et al. 2022; Zhang et al. 2021), and occipital (Zawiślak-Fornagiel et al. 2023b) areas in cognitively impaired PD patients relative to PD-NC or HC. PD patients whose cognitive status worsened at a 2- and 4-year follow-up evaluation exhibited higher theta band activity in frontal, temporal, parietal and occipital regions than those whose cognitive status remained stable (Puig-Davi et al. 2024). One study reported lower theta band activity in PD-MCI compared to PD-NC in bilateral frontal, temporal, and right occipital regions (Elkholy et al. 2023). Moreover, in another study, higher theta band activity in frontal regions was observed only in PD patients with fronto-striatal deficits and not in those with posterior-cortical deficits or with normal cognition (Betrouni et al. 2022).

*Beta* band activity was reportedly lower in PD patients exhibiting cognitive impairment relative to PD-NC or HC in temporal (Abbood et al. 2024; Elkholy et al. 2023; Zawiślak-Fornagiel et al. 2023b), frontal (Abbood et al. 2024; Elkholy et al. 2023), parietal (Zawiślak-Fornagiel et al. 2023b), occipital (Elkholy et al. 2023; Zawiślak-Fornagiel et al. 2023b) and central (Abbood et al. 2024) regions. High-beta activity was lower in PD patients with a fronto-striatal subtype of MCI compared to patients with a posterior-cortical subtype and PD-NC (Betrouni et al. 2022).

#### Relationship with cognitive assessment

Ten studies examined the relationship between spectral features and performance on cognitive assessments. Overall, increased slow-wave activity was associated with poorer performance across a range of cognitive domains.

Global cognition, as measured by the Montreal Cognitive Assessment (MoCA), positively correlated with alpha and beta band activity in frontal, temporal and occipital regions (Elkholy et al. 2023), in central and parietal regions (Anjum et al. 2024), as well as with the alpha/theta ratio predominantly in parietal regions (Anjum et al. 2024) and low alpha activity in the default mode network (DMN) (Yassine et al. 2023). Alpha activity was similarly positively correlated with Mini-Mental State Examination (MMSE) scores (Yılmaz et al. 2020), while absolute frontal delta power (Liu et al. 2022) was inversely associated with MMSE performance. Moreover, baseline theta band activity in frontal, central, temporal and parietal regions correlated negatively with PD-Cognitive Rating Scale (PD-CRS) scores at a 2-year follow-up assessment (Puig-Davi et al. 2024).

Memory performance was positively associated with beta (Anjum et al. 2024) and alpha band activity (Yılmaz et al. 2020) and with the alpha/theta ratio (Orso et al. 2022) in parietal regions, and negatively with theta activity in central and temporal regions (Anjum et al. 2024). Moreover, immediate and delayed recall memory performance positively correlated with the [alpha + beta]/[delta + theta] power ratio in frontal, central, temporal, parietal and occipital regions (Guner et al. 2017). Delayed recognition, however, was negatively associated with the power spectral ratio in all explored regions. Executive functions were positively associated with alpha and beta band activity in parietal regions (Anjum et al. 2024), with DMN-low alpha activity (Yassine et al. 2023) as well as with EEG median frequency (Zimmermann et al. 2015), and were negatively associated with theta band activity in central and temporal regions (Anjum et al. 2024). In one study, however, performance on tasks of executive function was negatively associated with the [alpha + beta]/[delta + theta] spectral power ratio in central, temporal, parietal and occipital regions (Guner et al. 2017). Visuospatial and visuoconstructional abilities were positively associated with the alpha/theta ratio in parietal (Eichelberger et al. 2017; Jaramillo-Jimenez et al. 2021; Orso et al. 2022;) and occipital regions (Eichelberger et al. 2017; Jaramillo-Jimenez et al. 2021) and with the [alpha + beta]/[delta + theta] power ratio in central, temporal, parietal and occipital regions (Guner et al. 2017). Language abilities were positively correlated with alpha and beta band activity in parietal regions (Anjum et al 2024), alpha band activity (Yılmaz et al. 2020) and with the [alpha + beta]/[delta + theta] power ratio in frontal, central, temporal, parietal and occipital regions (Guner et al. 2017). Processing speed correlated positively with beta band activity in parietal regions and negatively with theta band activity in central and temporal regions (Anjum et al. 2024).

### Source localisation and cognitive manifestations

Fifteen studies employed source localisation in their analyses (Table 4). Five used an MNE method to solve the inverse problem (Betrouni et al. 2022; Cai et al. 2021; Conti et al. 2023; Hassan et al. 2017; Yassine et al. 2023), and 10 used LORETA (Babiloni et al. 2019; Mostile et al. 2019; Pal et al. 2021; Puig-Davi et al. 2024).

**Table 4:** Summary of methodological choices and main outcomes from source localisation

Study	EEG parameters	Method/solution to inverse problem	Main findings	
			Source localisation	Correlations with cognitive assessments
Babiloni et al. (2017)	EC, 19 ch 5 min, –	eLORETA	**Low-α** (P, O, T, L): AD-MCI < PD-MCI < HC; **high-α** (P, O, T, L): AD-MCI < PD-MCI < HC; **δ** (P): PD-MCI > AD-MCI > HC	Negative correlation between MMSE score and delta (P) source activity. Positive correlation between MMSE score and **low-α** (P, O, T, L). Positive correlation between MMSE score and **high-α** (P, O, T, L)
Babiloni et al. (2018a)	EC, 19 ch, 5 min, –	eLORETA	Source localisation used to reconstruct cortical signals before computing FC. See Table 5 for results	–
Babiloni et al. (2018b)	EC, 19 ch, 5 min, –	eLORETA	Source localisation used to reconstruct cortical signals before computing FC. See Table 5 for results	–
Babiloni et al. (2019)	–, 20 ch, –, –	eLORETA	**δ**: PD-MCI, PDD > PD, HC (F, C, P, T, O, L); **low-α**: PD-MCI, PDD < PD, HC (O); **high-α**: PDD > PD-MCI > PD (T); **high-α**: PDD > PD-MCI, PD (F, C, T)	Negative correlation between MMSE score and widespread **δ** source activities (F, C, P, T, O, L)
Babiloni et al. (2020)	EC, 19 ch, 5 min, OFF/ON	eLORETA	**Low-α** (F, P, T, L): PD-MMSE+ > PD-MMSE−	–
Babiloni et al. (2024)	EO, 30 ch, 1 min, –	eLORETA	**α reactivity** (P): HC > ADD > PDD; **α reactivity** (O): HC > ADD, PDD; **α reactivity** (C): HC > PDD	Negative correlation between MMSE score and **α** source reactivity (C, P, O) to eyes open from eyes closed condition
Betrouni et al. (2022)	EC, 128 ch, 10 min, –	wMNE	Source localisation used to reconstruct cortical signals before computing FC; no direct source-level spectral power changes reported. See Table 5 for results	–
Bin Yoo et al. (2018)	EC, 19 ch, 5 min, –	sLORETA	Regional activity: **low-α** (bilateral posterior parietal cortex, frontal eye field [top-down stream]): PD > HC; **high-α** (bilateral posterior parietal cortex, frontal eye field [top-down stream]): PD > HC; **γ** (temporo-parietal junction, ventrolateral prefrontal cortex [bottom-up stream]): PD > HC. Functional connectivity: **high-α** (bilateral posterior parietal cortex, frontal eye field [top-down stream]): PD > HC	No correlation with UPDRS I scores
Cai et al. (2021)	EC/EO, 64 ch, 8 min, –	MNE	Source localisation used to reconstruct cortical signals before computing FC; no direct source-level spectral power changes reported. See Table 5 for results	–
Conti et al. (2023)	EC, 64 ch, 5 min, –	wMNE	Source localisation used to reconstruct cortical signals before computing FC; no direct source-level spectral power changes reported. See Table 5 for results	–
Hassan et al. (2017)	EC, 128 ch, 10 min, –	wMNE	Source localisation used to reconstruct cortical signals before computing FC; no direct source-level spectral power changes reported. See Table 5 for results	–
Mostile et al. (2019)	EC, 19 ch, –, –	sLORETA	**α** (BA17): PD-MCI < PD-NC; **β** (BA7): PD-MCI < PD-NC; **θ, δ** (BA11): PD-MCI > PD-NC; **δ** (BA7): PD-MCI < PD-NC	–
Pal et al. (2021)	EC, 128 ch, 3 min, –	sLORETA	**Microstate Class D** (current density in precuneus, cuneus and superior parietal lobe): PD-NC > HC; No significant difference in cortical source activity of **Microstate Class C** in any groups	–
Puig-Davi et al. (2024)	EC, 19 ch, 8 min, –	sLORETA	**θ, δ** (superior temporal gyrus, middle temporal gyrus, anterior pole of medial temporal region, fusiform gyrus, parahippocampal gyrus, inferior parietal lobe, precuneus, middle frontal region, inferior frontal regions): PD-progressors > PD-stable	Negative correlation between PD-CFRS score at 4-year follow-up and widespread **δ** and **θ** source activities (F, P, T, O). Negative correlation between PD-CRS at 2-year follow-up and **θ** activity (F, P, T, C). Negative correlation between visuospatial performance at 2-, 3- and 4-year follow-up and baseline **θ** and **δ** activity in left fusiform gyrus and posterior cingulate cortex. Negative correlation between language performance at 2- and 4-year follow-up and baseline left inferior parietal **θ** and **δ** activity. Negative correlation between delayed recall memory performance at 4-year follow-up and baseline left mid temporal, left mid frontal and left inferior frontal **θ** and **δ** activity
Yassine et al. (2023)	EC, 256 ch, 12 min, –	wMNE	Source-reconstructed signals were used to estimate the power spectrum at the cortical level using the Welch method, and relative band power features were estimated in five different EEG frequency bands. See sections “Spectral characteristics of cognitive manifestations”, “Functional connectivity characteristics and cognitive manifestations” and “Prediction and classification of cognitive outcome” for results	–

If studies reported the number of electrodes rather than the number of channels, a one-to-one correspondence was assumed and reported in the table unless otherwise specified

*EC* eyes closed, *EO* eyes open, *ch* channels, *HC* healthy controls, *PD* Parkinson’s disease, *PD-NC* Parkinson’s disease with normal cognition, *PD-MCI* Parkinson’s disease with mild cognitive impairment, *PDD* Parkinson’s disease with dementia, *PD-ND* Parkinson’s disease without dementia, *PD-MMSE+/− *Parkinson’s disease with low/high MMSE score (both groups include both PD-MCI and PD-NC) defined by Babiloni et al. (2020), *DLB* dementia with Lewy bodies, *AD* Alzheimer's disease, *AD-MCI* Alzheimer’s disease with mild cognitive impairment, *ADD* Alzheimer’s disease with dementia, *EEG* electroencephalography, *FC* functional connectivity, *F* frontal, *T* temporal, *P* parietal, *C* central, *O* occipital, *L* limbic, *MNE* minimum norm estimate, *LORETA* low resolution electrical tomography, *MMSE* Mini Mental State Examination, *UPDRS* Unified Parkinson’s Disease Rating Scale, *CRS* Cognitive Rating Scale, *CFRS* Cognitive Functional Rating Scale

Relative to PD-NC, PD-MCI patients had decreased alpha activity in the lingual gyrus, increased beta activity over the frontal lobe coupled with decreased activity in the precuneus, reduced theta and delta activity in the postcentral gyrus, and elevated theta and delta activity in the superior and middle frontal gyri, respectively (Mostile et al. 2019). Both low- and high-alpha source activities in parietal, occipital, temporal and limbic regions were lower in PD-MCI than in HC (Babiloni et al. 2017), and amplitude reduction of rsEEG alpha rhythms in the transition from the eyes-closed to -open conditions (i.e., alpha *reactivity*) was lower in PDD than in HC in parietal, occipital and central regions (Babiloni et al. 2024). Moreover, PD patients showing progressive cognitive decline were found to have higher theta and delta activity in the superior and middle temporal gyri that extended to the anterior pole of the medial temporal region, the fusiform and parahippocampal gyri, the inferior parietal lobe, the precuneus and the middle and inferior frontal regions compared to PD patients with a relatively stable cognitive status over a 4-year period (Puig-Davi et al. 2024).

One study comparing PD patients with more varied degrees of cognitive deficits reported a discriminant pattern in frontal, central, parietal, temporal, occipital and limbic delta source activities, such that PD-MCI and PDD patients had more activity than PD-NC and HC (Babiloni et al. 2019). In the same study, occipital low-alpha source activities were higher in HC and PD-NC than in PD-MCI and PDD. High-alpha sources, however, showed greater magnitude as a function of global cognitive impairment; temporal high-alpha source activities were greater in PDD than in PD-MCI, and in PD-MCI than in PD-NC. Moreover, frontal, central and temporal high-alpha source activities were greater in PDD than in both PD-MCI and PD-NC. Similar results were observed in a later study by Babiloni et al. (2020), who reported greater frontal, parietal, temporal and limbic low-alpha source activity in PD patients with high cognitive deficits relative to those with low cognitive deficits. Moreover, both low- and high-alpha source activities were reportedly greater in bilateral posterior parietal regions in PD-NC relative to HC, while gamma source activity was higher in the temporo-parietal junction and ventrolateral prefrontal cortex of the PD-NC group (Bin Yoo et al. 2018). One study on EEG microstates reported a significant difference in the cortical source activity underlying class D between PD-NC and HC, such that a pattern of higher current density in the precuneus, cuneus and superior parietal lobe characterised the PD-NC group (Pal et al. 2021).

In relation to cognitive performance, negative correlations were reported between baseline delta and theta activity in both the left fusiform gyrus and posterior cingulate cortex with visuospatial abilities at a 2- and 4-year follow-up (Puig-Davi et al. 2024). Baseline activity in the left inferior parietal region was similarly negatively correlated with language performance at each follow up, and negative correlations were found between baseline activity in left middle temporal, left medial frontal, and inferior frontal regions and memory performance (Puig-Davi et al. 2024). In another study, a statistically significant negative correlation was reported between widespread delta source activity and MMSE score when PD-NC, PD-MCI and PDD patients as well as HC were considered as a whole group (Babiloni et al. 2019). Similar results were reported when only the patients were considered a group (Babiloni et al. 2019). Moreover, MMSE scores correlated negatively with parietal delta source activity (Babiloni et al. 2017) and with alpha reactivity to eyes opening in central, parietal and occipital regions (Babiloni et al. 2024), and correlated positively with both low- and high-alpha sources in parietal, occipital, temporal and limbic regions (Babiloni et al. 2017).

### Functional connectivity characteristics and cognitive manifestations

#### Functional connectivity characteristics

Twenty-four studies evaluated EEG-based FC in relation to cognitive impairment in PD (Table 5). Overall, cognitive decline in PD was associated with widespread FC disruptions, with specific patterns of altered connectivity observed across different frequency bands and brain regions.

**Table 5:** Summary of methodological choices and main functional connectivity and microstate outcomes

Study	EEG parameters	FC measures	Main findings	
			Group comparison	Correlation with cognitive assessment
Babiloni et al. (2018a)	EC, 19 ch, 5 min, –	Linear lagged connectivity	**Low-** and **high**-**α**, interhemispheric in P T O lobes: PDD < HC. **High-α**, interhemispheric in P T O lobes: PDD > DLB. **High**-**α** intrahemispheric in F C P O T: PDD < HC. **Low-α** intrahemispheric in C P O T: PDD > DLB	No correlation when just PDD is considered
Babiloni et al. (2018b)	EC, 19 ch, 5 min, –	Linear lagged connectivity	**Low-** and **high-α**, interhemispheric in O T P lobes: PD-MCI < HC. Global **low-** and **high-α**, intrahemispheric: PD-MCI < HC	No correlation when just PD-MCI is considered
Betrouni et al. (2022)	EC, 128 ch, 10 min, ON	PLV	**High-β** pairwise lobe connections (F-P, F-L, T-P, T-L, P-L, O-L): PD-FS < PD-NC, PD-FS < PD-PC	–
Cai et al. (2021)	EC/EO, 64 ch, 8 min, –	dwPLI	**θ**: brainwide PD-MCI > PD-NC	MoCA negative correlation with dWPLI in left and right PMFG. Poorer attention function related to higher left PMFG connectivity, poorer visuospatial function and attention function related to higher right PMFG connectivity
Carmona Arroyave et al. (2019)	EC, high density, 15 min, –	Coherence	**High-α** intrahemispheric F-T left and F-P (l): PD-NC < HC; **High-α** intrahemispheric F-P (l) and (r): PD-MCI > PD-NC. **High-α** interhemispheric O–O and P-P: PD-NC < HC, **low-β** interhemispheric F-F: PD-MCI < PD-NC, interhemispheric P-P: PD-MCI > PD-NC. **Low** and **high-β** intrahemispheric F-F: PD-MCI < PD-NC	IFS score positive correlation with **high-β** in right frontal ROI in PD-NC. IFS score negative corr with **high-β** in left frontal ROI in PD-MCI
Chaturvedi et al. (2019)	EC, 256 ch, 12 min, –	PLI	**θ**: PD-MCI > PD-NC	Memory, working memory and attention negative correlation with **θ**. Visuospatial function positive correlation with **low-α**. Memory positive correlation with **high-α**. Working memory and attention positive correlation with **β**
Chu et al. (2023a)	EC, –, 5 min, –	Entropy, Energy, Occurrence, Duration	Log(Energy) in subnetwork 1, 2, 3: PD-MCI < PD-NC. Entropy in subnetwork 1,2,3: PD-MCI < PD-NC. Occurrence in subnetwork 1: PD-MCI < PD-NC and in subnetwork 4, 5 PD-MCI > PD-NC. Duration in subnetwork 1, 3: PD-MCI < PD-NC, and in subnetwork 4, 5: PD-MCI > PD-NC	MoCA positive correlation with entropy in all five subnetworks. MoCA positive correlation with Log(Energy) in subnetworks 1,2,3. MoCA positive correlation with occurrence in subnetwork 1 and negative correlation in subnetwork 4,5. MoCA positive correlation with duration in subnetwork 1,3 and negative correlation in subnetwork 4, 5
Conti et al. (2023)	EC, 256 ch, 10 min, –	PLI, Network based statistic (NBS)	**θ** (L, T, P, O): PD < HC. **α** (P, F, T, L): PD < HC. **β** (F, L): PD > HC	Total NMSS negative correlation with **θ** in F-L, T-L. NMSS domain 3 (mood/cognition) negative correlation with θ in T-P, F-T. NMSS domain 5 negative correlation with **θ** (attention/memory) in F-O, T-P
Conti et al. (2025)	EC, 64 ch, 5 min, –	wPLI, Network based statistic (NBS)	mNC **α** network (left superior frontal and bilateral pars opercularis, bilateral paracentral and right precentral cortices, bilateral superior temporal cortex): PDpRBD+ < PDpRBD− < HC; mNC **β** network (left precentral, bilateral postcental cortices, bilateral anterior and posterior cingulate cortex, right precuneus) PDpRBD+ > HC, PDpRBD− > HC. Asymmetry index **β** network: PDpRBD+ < PDpRBD−	MoCA positive correlation with mNC **α** network in both PDpRBD+ and PDpRBD−
Elkholy et al. (2023)	EC, 19 ch, 3–4 min, –	Coherence	**δ**, **α** intrahemispheric F-P left, **θ** interhemispheric F: PD-MCI differ from PD-NC	–
Hassan et al. (2017)	EC, 128 ch, 10 min, –	PLV, graph measures	**High-α** PLV in F-T: PD-MCI < PD-NC; **high-α** PLV in F–C, T-F, F-P, O-C: PDD < PD-MCI; **high-α** PLV in T-F, F–C, F-T: PDD < PD-NC. **Low-α** PLV in T-F, T-T, F–C: PDD < PD-MCI; **low-α** PLV in T-F, F–C, T-T: PDD < PD-NC	Composite cognitive score positive correlation with edgewise connectivity index
Iyer et al. (2020)	EC, 128 ch, 3 min, ON	dwPLI, NBS	**θ** and **γ** connectivity in F-T, F-P: PD > HC	Higher **γ** connectivity between fronto-temporal regions and higher **θ** connectivity within frontal and parietal networks correspond to higher cognitive impairment and anxiety
Jeong et al. (2015)	EC, 21 ch, 10 min	Wavelet energy (RWE) and coherence (WC)	RWE **β** P, F, O: PDD < HC. RWE **θ** O: PDD > HC. WC **α** in F7-T5, Fp2-T3, F8-T5: PDD > AD, in F8-T6: PDD > HC. WC **β** in T3-CZ, T4-C3, T6-C3, F3-O2: PDD > AD, PDD > HC; in F7-PZ, T5-PZ PDD > HC. WC **γ** in F7-T5, Fp2-T3, F8-T5: PDD > AD, in T4-C3, T6-C3 PDD > HC	–
Mano et al. (2022)	EC, 15 ch, –, ON	Coherence	Whole brain and l- F-F, F-T, F-P, F-P, r- F-P: PD-MCI < HC-MCI	HDS-R—global, l- F-T, F-P, F-P. FAB—global, l- T-T, P-O, F-P, F-P, F-P (r). MMSE—global, r- F-F,T-T, F-T, P-O, F-P
Mehraram et al. (2020)	EO, 128 ch, 2.5 min, –	wPLI, graph measures	wPLI: **α** PDD < HC, **β** PDD < HC, PDD < AD. Node degree: **α** PDD < HC, **β** PDD < AD. Clustering coefficient: **α** PDD < HC, **β** PDD > DLB. Characteristic path length: **α** PDD > HC, PDD > AD. Small worldness: **θ** PDD > AD. Modularity: **θ** PDD > HC, PDD > AD, **α** PDD > HC, PDD > AD, **β** PDD > AD	Considering PDD: animal naming positive correlation with **α** characteristic path length; MMSE negative correlation with **θ** small worldness, CAF, NPI positive correlation with **θ** small worldness; MMSE negative correlation with **θ** modularity; CAF positive correlation with **θ** modularity
Peláez Suárez et al. (2021)	–, –, 30 min, ON	SL, Graph measures	**α**, **θ** SL: PD-NC < HC, PD-MCI < HC; **β** SL: PD-NC > HC, PD-MCI > HC; **α**, **δ** SL: PD-MCI < PD-NC. Clustering coefficient (**α, β**), l mean path length ( **θ, δ**): PD-MCI < HC. Mean path length (**θ, β**): PD-NC < HC. Global connectivity (**β**): HC < PD-NC. Local efficiency (**θ, β**): PD-NC < HC	FAB positive correlation **β** global connectivity. FAB negative correlation with **β** path length. WAIS III negative correlation with **β** path length
Peraza et al. (2018)	EC, 128 ch, 2.5 min, –	PLI, Minimum spanning tree	Degree: **α** PDD < HC. Leaf **α** PDD < HC. Diameter, eccentricity and radius: **high-θ** PDD > HC, PDD > AD. PLI: **θ** PDD > HC, **high-θ** PDD > HC, PDD > AD, **α** PDD < HC, PDD < DLB, **β** PDD < HC, PDD < AD. Root: **θ** PDD > HC, **high**-**θ** PDD > HC, PDD > AD, **α** PDD < HC PDD < DLB, **β** PDD < HC, PDD < AD. Leaf: **θ** PDD > HC, **high**-**θ** PDD > HC, PDD > AD, **α** PDD < HC. Height: **high-θ** PDD > HC, **α** PDD < HC, PDD < DLB, **β** PDD < HC	No correlation when just PDD considered
Sánchez-Dinorín et al. (2021)	EC, 52 ch, 2 min, –	PLV	**δ**, **θ**, **α**, **β**, **γ** in frontal regions: PD > HC	CASI and visuospatial domain negative correlation with frontal **δ** and **θ** for disease duration > 8 years. CASI positive correlation with frontal **δ** and **θ** when disease duration < 3 years and < 2 years respectively. Visuospatial domain weak positive correlation with frontal **δ** when disease duration < 1 year
Teramoto et al. (2016)	EC, 16 ch, –, –	Coherence	F3-P3 and F4-P4 **α** coherence have a low-coherence and a high-coherence group. PD with executive dysfunction tend to fall in the low coherence groups	Lower standardized BADS score in the low F3-P3 coherence group
Utianski et al. (2016)	EC, 21 ch, –, ON	PLI, graph measures (weighted network, minimum spanning tree)	Clustering coefficient, characteristic path length, modularity (**all frequency bands**): PD > HC. **δ**, **low-α** degree divergence: PD < HC. **θ**, **β**, **high-α** degree divergence: PD > HC. **δ**, **θ** MST diameter PD < HC. **δ** MST eccentricity PD < HC. **δ**, **θ** MST leaf fraction: PD > HC. **low-α** PLI, clustering coefficient, characteristic path length, degree divergence, MST betweenness centrality, MST leaf fraction: PD > PDD. **δ** MST betweenness centrality, **high-α** Modularity and MST diameter and eccentricity, **low-α** modularity and MST diameter: PD < PDD. **low-α** clustering coefficient and degree divergence and MST leaf fraction: PD > PD-MCI. **δ**, **θ** PLI: PD < PD-MCI	MMSE positive correlation with: **low-α** PLI and clustering coefficient, **α** degree divergence, **δ** MST diameter and eccentricity. MMSE negative correlation with: **β** clustering coefficient, **δ** MST betweenness centrality, **high-α** MST diameter, **high-α** MST eccentricity. MoCA positive correlation with: **α** clustering coefficient, **δ** and **α1** degree divergence, **θ** and **low-α** MST leaf fraction. MoCA negative correlation with: **δ** and **low-α** Modularity, **θ** and **low-α** MST eccentricity
Yassine et al. (2023)	EC, 256 ch, 12 min, –	Amplitude Envelope Correlation (AEC)	See Table 3 for results	See Table 6 for results
Yi et al. (2022)	EC, 19 ch, –, –	PLI, Graph measures	**δ**, **θ**, **α** PLI: PD-MCI > PD. **α** path length and δ small worldness: PD-MCI > PD. **δ** and **θ** temporal correlation coefficients, **θ** and **α** small worldness, functional connectivity strength: PD-MCI < PD. **δ**, **α** temporal variability of FC: PD-MCI > PD	–
Zawiślak-Fornagiel et al. (2023a)	EC, 19 ch, 20 min, –	Coherence, PLI	PLI** θ** in lO-lT, lO-rF, lP-lF, lO-rC: PDD > PD-MCI and PDD > PD-NC; in lP-lT: PDD > PD-MCI; in lO-rF, lO-rP, rT-rF,lO-rO: PDD > PD-NC. Coherence** θ** in rT-lF: PDD < PD-MCI, PDD < PD-NC; in rT-rF: PDD > PD-MCI and PDD > PD-NC. Coherence** α** in lO-rF, rO-lF: PDD < PD-MCI and PDD < PD-NC; in lO-rC, rP-lT, rF-lF: PDD < PD-NC;in lO-rC PD-MCI < PD-NC. Coherence** β** in lO-rF, lT-rC, lP-rF: PDD < PD-NC; in lT-lF: PD-MCI < PD-NC. Coherence** γ** in rT-rC: PD-MCI < PD-NCand PD-MCI < PDD; in lO-rF, lP-rF: PDD < PD-NC; in lT-lF: PD-MCI < PD-NC	Lower ACE-III scores related to lower coherence in **α** and higher coherence in **θ**
Zawiślak-Fornagiel et al. (2023b)	EC, 19 ch, 20 min, ON	PLI	**θ** PLI in lP-rF, lO-rF, lO-lT: PDD > PD-NC. **θ** PLI in lP-lT, lO-lT: PD-MCI < PDD	–
Costa et al. (2023)	EO/EC, 32 ch, 5 min, ON	Microstates	Duration in **C** with EC: PD < HC	MMSE negative correlation with coverage of **C** in EO/EC. Verbal fluency negative correlation with coverage of **C** in EC. MMSE and verbal fluency positive correlation with coverage of **D** in EC
Chu et al. (2020)	EC, 19 ch, –, OFF	Microstates	MMD in **A**: PD > HC. OPS in **A**: PD > HC. OPS in **C**: PD < HC. RTC in **B**: PD > HC RTC in **C**: PD < HC	MoCA positive correlation with OPS of **C**. MoCA negative correlation with MDD of **B**
Chu et al. (2023b)	EC, 19 ch, 15–20 min, –	Microstates	Temporal variability in **A**: PD < HC rF, lP, PD > HC rF, lP. Temporal variability in **B**: PD > HC whole brain, lF, lP, lO. Temporal variability in **C**: PD < HC whole brain, lF, rO. Temporal variability in **D**: PD > HC in lF, rP	MoCA positive correlation with temporal variability of **C**. MoCA positive correlation with spatial variability of **D** in lT
Liu et al. (2023)	EC, 19 ch, 15 min, –	Microstates	MMD in **C**: PD-MCI < PD-NC. RTC in **A**,**D**: PD-MCI > PD-NC. RTC in **C**: PD-MCI < PD-NC. OPS in **A**, **D**: PD-MCI > PD-NC. OPS in **C**: PD-MCI < PD-NC	MoCA negative correlation with RTC and OPS of **A, D**. MoCA positive correlation with MMD, RTC, OPS of **C**
Pal et al. (2021)	EC, 128 ch, 3 min, –	Microstates	TF in **D**: HC > PDD, HC > PD-NC. TF in **C**: HC > PD-NC	–

When studies reported the number of electrodes rather than the number of channels, we assumed a one-to-one correspondence unless otherwise specified

*EC* eyes closed, *EO* eyes open, *ch* channels, *HC* healthy controls, *PD* Parkinson’s disease, *PD-MCI* Parkinson’s disease with mild cognitive impairment, *PDD* Parkinson’s disease with dementia, *DLB* dementia with Lewy bodies, *AD* Alzheimer’s disease, *F* frontal, *T* temporal, *P* parietal, *C* central, *O* occipital, *L* limbic, *r* right, *l* left, *PMFG* posterior division of the middle frontal gyrus, *MoCA* Montreal Cognitive Assessment, *MMSE* Mini Mental State Examination, *IFS* INECO frontal screening test, *NMSS* Non-Motor Symptoms Scale, *HDS-R* Revised Hasegawa Dementia Score, *FAB* Frontal Assessment Battery, *CAF* cognitive assessment of fluctuation, *NPI* neuropsychiatric inventory test subscale for the severity and frequency of hallucinations, *WAIS* Wechsler Adult Intelligence Scale, *CASI* Cognitive Abilities Screening Test, *ACE* Addenbrooke’s Cognitive Examination, *PDpRBD+/−* Parkinson’s Disease patients with rapid eye movement sleep behaviour disorder before/after parkinsonism, *PLI* phase lag index, *PLV* phase locking value, *mNC* mean network connectivity, *MST* minimum spanning tree, *MMD* mean microstate duration, *OPS* occurrence per second, *RTC* ratio of time coverage, *TF* time frame

Several studies reported increased connectivity in slower frequency bands in PD with cognitive impairment. In particular, increased whole-brain theta connectivity, as measured by PLI, was observed both in PD compared to HC (Iyer et al. 2020), and in PD-MCI compared to PD-NC (Cai et al. 2021; Chaturvedi et al. 2019; Utianski et al. 2016; Yi et al. 2022). A similar finding was reported for the delta band (Utianski et al. 2016; Yi et al. 2022). Regarding regional connections, increased theta PLI was observed in PD-MCI compared to PD-NC between the left parietal and right frontal regions (Zawiślak-Fornagiel et al. 2023b). When a group with dementia was included, PDD patients exhibited higher intra-hemispheric theta connectivity in the left occipito-temporal and left occipito-frontal regions, as well as greater interhemispheric connectivity between the left parietal and right frontal regions and between the left occipital and right central regions, compared to both PD-MCI and PD-NC (Zawiślak-Fornagiel et al. 2023a).

Conversely, connectivity in the alpha and beta bands was generally reduced in PD with cognitive impairment. Both left and right fronto-parietal alpha coherence was lower in the PD group with executive dysfunctions (Teramoto et al. 2016). PD-MCI patients exhibited lower fronto-frontal interhemispheric and intrahemispheric coherence than PD-NC in both low- and high- beta frequency (Carmona Arroyave et al. 2019). Decreased high-beta connectivity was observed in multiple large-scale networks, including fronto-parietal, fronto-limbic, temporo-parietal, temporo-limbic, parieto-limbic, and occipito-limbic connections, in PD with fronto-striatal cognitive deficits, compared to both PD-NC and PD with posterior-cortical cognitive impairments (Betrouni et al. 2022). A study using the presence of rapid eye movement (REM) sleep behaviour disorder before parkinsonism (pRBD) to distinguish between body-first (pRBD+) and brain-first (pRBD−) PD subtypes found that, compared to both HC and PD-pRBD−, PD-pRBD+ have lower mean network connectivity in the alpha network in bilateral pars ocularis, paracentral and superior temporal cortices, as well as in right precentral cortex; however, connectivity in the beta network was higher in both PD subtypes compared to HC (Conti et al. 2025). A study using PLV found reduced high-alpha connectivity in fronto-temporal, fronto-central, and occipito-central connections in cognitively impaired PD patients compared to PD-NC (Hassan et al. 2017). Moreover, PD-MCI patients exhibited lower SL in the alpha band than both HC and PD-NC (Peláez Suárez et al. 2021). When a group with PDD was included, these patients showed lower PLI alpha and beta connectivity compared to HC (Mehraram et al. 2020), and lower PLI low-alpha connectivity (Utianski et al. 2016), as well as reduced occipito-frontal alpha coherence compared to PD-MCI and PD-NC, and generally lower interhemispheric alpha and beta coherence compared to PD-NC (Zawiślak-Fornagiel et al. 2023a). Similar results were obtained using LLC in the high alpha band, as PD-MCI and PDD have lower values than HC in inter- and intra-hemispheric parietal, temporal and occipital lobes (Babiloni et al. 2018a, b).

Graph theoretical analyses revealed that PD-MCI patients exhibited a lower clustering coefficient and local efficiency in the beta and alpha bands, as well as increased path length in the beta, theta, and delta bands, relative to HC (Peláez Suárez et al. 2021). Additionally, PD-MCI patients demonstrated increased path length and greater temporal variability of FC in the alpha band, as well as greater small-worldness in the delta band, compared to PD patients (Yi et al. 2022). In contrast, PD-MCI exhibited lower temporal correlation coefficients in the delta and theta bands, along with reduced small-worldness and functional connectivity strength in the theta and alpha bands. Furthermore, PDD exhibited higher low-alpha modularity than PD-NC (Utianski et al. 2016). Additionally, data-driven dynamic subnetworks based on alpha PLI revealed distinct patterns of occurrence and duration in PD-MCI compared to PD-NC, with reduced values for Subnetwork 1 (temporal, parietal, occipital regions), but increased for Subnetwork 4 (temporal, parietal, frontal regions) (Chu et al. 2023a).

#### Relationship with cognitive assessment

Studies examining the relationship between FC measures and cognitive performance reported significant associations. Namely, frontal delta connectivity negatively affected global cognition, as assessed by the Cognitive Abilities Screening Test (CASI), and visuospatial function for disease duration > 8 years but showed a weak positive effect when disease duration was < 1 year (Sánchez-Dinorín et al. 2021). Increased theta coherence was associated with lower scores in the Addenbrooke’s Cognitive Examination (ACE-III) (Zawiślak-Fornagiel et al. 2023a). Moreover, increased fronto-parietal theta PLI positively correlated with PD-CRS and anxiety in PD-NC (Iyer et al. 2020). Frontal theta connectivity negatively affected CASI and visuospatial function for disease duration > 8 years; however, it had a positive effect on CASI when disease duration was < 2 years (Sánchez-Dinorín et al. 2021). Additionally, higher theta PLI negatively correlated with MoCA score (Cai et al. 2021) and, more specifically, with memory, attention, working memory and visuospatial functions (Chaturvedi et al. 2019; Cai et al. 2021). In PDD, theta small worldness and modularity correlated negatively with MMSE (Mehraram et al. 2020). Conversely, edgewise connectivity index in alpha positively correlated with a composite cognitive score (i.e., including attention and working memory, executive functions, episodic memory, language, visuospatial functions) (Hassan et al. 2017), and alpha PLI positively correlated with MMSE (Utianski et al. 2016). Similarly, mean network connectivity in the alpha network positively correlated with MoCA in both body-first and brain-first subtypes of PD (Conti et al. 2025). Moreover, higher low-alpha connectivity positively correlated with visuospatial function, while higher high-alpha connectivity positively correlated with memory (Chaturvedi et al. 2019). Lower alpha coherence was associated with lower ACE-III scores (Zawiślak-Fornagiel et al. 2023a) and executive dysfunction, assessed with the Behavioural Assessment of the Dysexecutive Syndrome (Teramoto et al. 2016). Regarding FC in the beta frequency band, executive dysfunctions positively correlated with global connectivity in beta and negatively correlated with mean path length, and a similar negative correlation was found between working memory and mean path length (Peláez Suárez et al. 2021).

#### Functional connectivity with microstates

Studies employing microstates showed that individuals with early PD-MCI exhibited decreased duration, time coverage, and occurrence of microstate C compared to those with early PD-NC. Conversely, microstates A and D demonstrated a higher occurrence and time coverage in early PD-MCI than in early PD-NC (Liu et al. 2023). Pal and colleagues (2021) reported a higher occurrence of microstate D in HC compared to both PD-NC and PDD, as well as a higher occurrence of microstate C in PD-NC compared to PDD. Regarding correlations with global cognitive functioning, the occurrence per second and the ratio of time coverage of microstate A negatively correlated with MoCA scores, whereas these same parameters, along with the mean duration of microstate C, positively correlated with MoCA scores (Liu et al. 2023).

### Prediction and classification of cognitive outcome

Twenty-three of the included studies addressed the prediction and classification of cognitive outcomes in PD using EEG features (Table 6).

**Table 6:** Summary of methodological choices and main outcomes of machine learning

Study	EEG parameters	Model	Main findings		
			Most relevant features/predictors	Performance	Correlations with cognitive assessments
Anjum et al. (2024)	EO, 64 ch, 10 min, ON	Linear predictive coding	LEAPD indices	Spearman correlation > 0.4 (Leave-one-out cross validation) and > 0.6 (out of sample test)	LEAPD indices correlated with MoCA scores. The best-performing LEAPD frequency ranges were broad for central-parietal electrodes and focused on lower frequencies in PO7, O2, P4, and F4
Arnaldi et al. (2017)	EC, 19 ch, –, –	Logistic regression, linear discriminant analysis	Posterior mean frequency	Accuracy 82%	Lower posterior mean frequency best predicted cognitive worsening. Patients who developed dementia could be distinguished from cognitively stable ones but not from other cognitively worsened individuals. Higher posterior mean frequency was associated with longer survival
Babiloni et al. (2017)	EC, 19 ch, 5 min, –	Receiver operating characteristic	Source activities in **δ** P; **low-**, **high-α** P O T L; ratio between **δ** and both **low-** and **high-α**	AUC > 0.7 HC vs PD-MCI with delta P, **δ/α** ratio (P)	See Table 5 for results
Babiloni et al. (2018a)	EC, 19 ch, 5 min, OFF	Receiver operating characteristic	Lagged linear connectivity in **low-α** and **high-α**	PDD vs HC: accuracy 76% with temporal interhemispheric **low-α**, and accuracy 69% with temporal interhemispheric **high-α**	See Table 5 for results
Babiloni et al. (2018b)	EC, 19 ch, 5 min, OFF	Receiver operating characteristic	Lagged linear connectivity in **low-α**	PD-MCI vs HC: accuracy 68% with temporal interhemispheric **low-α**. No significant results for PD-MCI vs AD-MCI	See Table 5 for results
Babiloni et al. (2019)	–, 20 ch, –, –	Receiver operating characteristic	**δ** and **α** source activity	HC vs PD AUC = 0.74; HC vs PD-MCI AUC > 0.7; HC vs PDD AUC > 0.9; PD vs PD-MCI AUC > 0.7; PD vs PDD AUC > 0.85; PD-MCI vs PDD AUC > 0.7	HC vs. PD classification was possible only using **α** in T, while **δ** source activity in C, T, P, L regions allowed classification across all cognitive groups
Betrouni et al. (2019)	–, 128 ch, –, –	Dimensionality reduction, SVM Model, kNN Model	Peak frequency; absolute power in **θ**, **low**-**α**, **high**-**β**; relative power in **δ**, **θ**, **low-** and **high-α**, **low-β**	SVM cross validation accuracy 86%. kNN cross validation accuracy 87%	Classification into 5 cognitive subgroups (cognitively intact, mental slowing, mild cognitive deficits, severe deficits particularly in executive functions, severe deficits particularly in memory functions)
Cai et al. (2021)	EO/EC, 64 ch, 8 min, –	Logistic regression, receiver operating characteristic	PLI in left and right PMFG	AUC left = 0.77. AUC right = 0.81	Left and right PMFG were independent risk factors for MCI in PD, with higher connectivity predicting worse outcome
Chaturvedi et al. (2019)	EC, 256 ch, 12 min, –	Random forest	**θ** and **δ** PLI, **θ** relative power	Cross-validation AUC > 0.65. AUC on independent dataset > 0.74	Classification into PD-MCI and PD-NC, with **β** and **θ** PLI associated with memory functions
Choi et al. (2024)	EO/EC, –, 1 min, –	Linear regression	Relative **θ**, **α**, **β** power	Regression coefficients	Higher global relative **θ** power predicted lower MMSE, JLO, AVLT, STM, and LTM scores, as well as longer TMT-A/B times. Higher relative **β** power predicted higher JLO scores and shorter TMT-A/B times
Chu et al. (2023a)	EC, –, 5 min, –	Non-negative matrix factorisation	PLI on time-dependent sliding windows	Stability coefficient = 1 and stable reconstruction error gradient for a number of subnetworks equal to 5	–
Conti et al. (2025)	EC, 64 ch, 5 min, –	Receiver operating characteristic	**α** and **β** NBS networks	**α** network: accuracy 73% PD-pRBD− vsHC, 74% PD-pRBD− vsPD-pRBD+, 87%PD-pRBD+ vsHC. **β** network: accuracy 80% PD-pRBD− vsHC, 87% PD-pRBD+ vsHC	–
Cozac et al. (2016a, b)	EC, 256 ch, –, –	Linear regression, receiver operating characteristic, random forest	Global relative median power in **θ**	25% explained variance, AUC = 0.75	Global relative median power in **θ** was one of the best predictors of cognitive worsening at a 3-year follow-up in linear regression and the most relevant feature in Random Forest classification of cognitive outcomes
Gschwandtner et al. (2023)	EC, 256 ch, 15 min, ON	Connectome-based predictive modelling	PLI, TBPC in **θ**, **δ**, **β**	PLI AUC 0.7, TBPC AUC 0.8 for HC vs PD	Higher baseline TBPC predicted worse future cognitive outcome
Jennings et al. (2022)	EO/EC, 128 ch, 2.5 min, –	kNN model	EC: C and F **θ**, O **δ**, F **β**, P **α**. EO: F **θ**, P **θ**, O **δ**	KNN (EC only): cross-validation accuracy 84.3% for HC vs Dementia, and cross-validation accuracy 96.4% for PDD vs AD/DLB. KNN (EC + EO): cross-validation accuracy 87.7% for HC vs Dementia, and cross-validation accuracy 96.8% for PDD vs AD/DLB	–
Jeong et al. (2015)	EC, 21 ch, 10 min,	Linear discriminant analysis	Wavelet energy and coherence	Wavelet energy: PDD vs HC accuracy 79%, PDD vs AD accuracy 74%. Wavelet coherence: PDD vs HC accuracy 79%, PDD vs AD accuracy 80%	–
Kozak et al. (2020)	EC, 256 ch, 15 min, –	Linear regression	Global **θ** power	10% explained variance	Higher baseline global **θ** power predicted cognitive decline at a 3-year follow-up
Latreille et al. (2016)	EC, 12 ch, 10 min, –	Logistic regression	Absolute spectral power, occipital frequency	Odds Ratio = 40.73 (95% CI 1.75–949.95) for occipital EEG slowing, odds ratio = 3.95 (95% CI 1.07–14.71) for dominant occipital frequency	Occipital EEG slowing and dominant **α** frequency (O) predicted dementia outcome in PD
Liu et al. (2022)	EC, 19 ch, 15 min, –	Receiver operating characteristic	**δ** power (F), combined with HDL-C and Hs-CRP markers	PDD vs PD-NC: AUC = 0.74 with only **δ** (F). AUC = 0.81 with **δ** (F) combined with HDL-C and Hs-CRP	See Table 4 for results
Liu et al. (2023)	EC, 19 ch, 15 min, –	Convolutional neural network	Power spectral density	Mean cross-validation accuracy 99%	Identified 1–11.5 Hz as the frequency band showing the largest difference between early PD-MCI and early PD-NC
Yassine et al. (2023)	EC, 256 ch, 12 min, –	Similarity network fusion	Power spectral density, functional connectivity	Robustness coefficient 91% on 100 iterations	Three clusters were identified. G1 and G2 showed stable EEG features at a 5-year follow-up, while G3 exhibited increased **δ** and **θ** power and slower **α** and **β** at both baseline and follow-up. The most relevant brain networks were SMN in **δ** and **β**, DMN in **low α**, and FTN in **high α**
Yi et al. (2022)	EC, 19 ch, –, –	SVM model	Complexity, FC strength, FC temporal variability	Accuracy 80%	Classified PD and PD-MCI
Zhang et al. (2021)	EO, 16 ch, 30 min, –	SVM model	qEEG features	Leave-one-out cross validation accuracy 70%	Classified PD-NC and PD-MCI. The most relevant qEEG features were **θ** waves in C3, T5, and P3 and **δ** waves in O2 and P3

When studies reported the number of electrodes rather than the number of channels, we assumed a one-to-one correspondence unless otherwise specified

*EC* eyes closed, *EO* eyes open, *ch* channels, *qEEG* quantitative EEG, *FC* functional connectivity, *HC* healthy controls, *PD* Parkinson’s disease, *PD-MCI* Parkinson’s disease with mild cognitive impairment, *PDD* Parkinson’s disease with dementia, *DLB* dementia with Lewy bodies, *AD* Alzheimer’s disease, *F* frontal, *T* temporal, *P* parietal, *C* central, *O* occipital, *L* limbic, *r* right, *l* left, *PMFG* posterior division of the middle frontal gyrus, *SMN* sensorymotor network, *FTN* fronto-temporal network, *DMN* default mode network, *MoCA* Montreal Cognitive Assessment, *MMSE* Mini Mental State Examination, *JLO* Judgment of Line Orientation, *AVLT* auditory-verbal learning test, *STM* short term memory, *LTM* long term memory, *TMT* Trail Making Test, *PDpRBD+/−* Parkinson’s disease patients with rapid eye movement sleep behaviour disorder before/after parkinsonism, *HDL-C* high-density lipoprotein cholesterol, *Hs-CRP* hypersensitive C-reactive protein, *LEAPD* linear-predictive-coding EEG Algorithm for PD, *NBS* network based statistic, *TBPC* time between phase crossing, *PLI* phase lag index, *PLV* phase locking value, *kNN* k-nearest neighbour, *SVM* support vector machine, *AUC* area under the curve, *CI* confidence intervals. G1, G2 and G3 are clusters of electrophysiological profiles, defined by Yassine and colleagues (2023) as a mild-to-severe (diffuse) group with progressive cognitive decline (G3) and moderate (motor only) groups with more rapid motor progression (G1, G2)

Prediction studies employed linear regression, logistic regression, linear discriminant analysis, and linear predictive coding. Lower posterior mean frequency was identified as the best predictor of cognitive worsening and was associated with lower survival time (Arnaldi et al. 2017). In the same study, patients who developed dementia could be distinguished from the cognitively stable group but not from other cognitively impaired individuals. Similarly, Latreille et al. (2016) found occipital EEG slowing to be the best predictor of dementia in PD. In addition, cognitive worsening at a 3-year follow-up, assessed using a composite cognitive score combining attention, memory, verbal fluency, working memory, executive and visuospatial functions, was predicted by higher theta power at baseline (Cozac et al. 2016a; Kozak et al. 2020). The analysis of individual cognitive domains revealed that higher global relative theta power predicted lower MMSE scores, and poorer visuospatial (i.e., Judgment of Line Orientation [JLO]), memory (i.e., Auditory Verbal Learning Test) and attention performance (i.e., Trail Making Test A and B [TMT-A/B]) (Choi et al. 2024). In the same study, higher relative beta power predicted better performance in the JLO and TMT-A/B. Moreover, time between phase crossing in delta, theta and beta frequencies at baseline as features for predictive modelling could predict cognitive worsening at 3-year follow-up (Gschwandtner et al. 2023).

Studies focusing on classification employed SVM, kNN, Random Forest, and Receiver Operating Characteristic (ROC). Global relative theta power achieved the best accuracy in the ROC curve analysis of overall cognitive scores, with an Area Under the Curve (AUC) of 75%, and was also the most important variable in classification using Random Forest (Cozac et al. 2016a). Additionally, Random Forest with relative theta power and theta PLI as features classified PD-NC and PD-MCI with an accuracy of 74% (Chaturvedi et al. 2019). Early PD-NC and early PD-MCI could be classified with EEG signal complexity, FC strength and temporal variability with an accuracy of 80%, employing an SVM model (Yi et al. 2022). With the same classifier, Betrouni et al. (2019) reported that relative and absolute theta power were consistently selected in a dimensionality reduction procedure across all used models for classifying PD patients into five cognitive subgroups. Considering all qEEG features, theta waves in central and temporal regions were the most significant SVM classification features for distinguishing PD-NC from patients with cognitive impairment (Zhang et al. 2021). By contrast, Babiloni et al. (2019) used ROC with delta source activity in central, parietal, temporal, and limbic regions to perform pairwise classification between HC, PD-NC, PD-MCI, and PDD. Similarly, delta power in frontal regions could classify PDD and PD-NC with an AUC of 0.74, which improved to 0.81 when adding hypersensitive C-reactive protein (Hs-CRP) and high-density lipoprotein cholesterol (HDL-C) as features (Liu et al. 2022). When given interhemispheric high-alpha as a feature, a ROC model achieved 68% and 82% accuracy in labelling HC versus PD-MCI and PDD respectively (Babiloni et al. 2018a, b). Alpha functional networks can characterise PD subtypes, as they classify HC, PD-pRBD+ and PD-pRBD− with an accuracy higher than 70% (Conti et al. 2025). Additionally, parietal delta and its ratio with high-alpha could classify HC and PD-MCI with an AUC higher than 0.7 (Babiloni et al. 2017).

Unsupervised learning was performed using convolutional neural networks and similarity network fusion. When trained on power spectral density data computed from 1 to 45 Hz in 0.5 Hz increments across 19 EEG channels, the CNN identified the frequency range of 1 to 11.5 Hz as the most relevant in classifying between early PD and early PD-MCI (Liu et al. 2023). Similarity network fusion identified three distinct subgroups based on power spectral and FC features. Two of these clusters were more similar to each other, both primarily characterised by motor-related symptoms, while the third cluster comprised subjects with more diffuse symptoms and lower MoCA scores. This cluster exhibited increased delta and theta frequencies and reduced alpha and beta frequencies. The functional networks that best differentiated the clusters were the sensorimotor network (SMN) in delta and beta, the DMN in low-alpha, and the fronto-temporal network (FTN) in high-alpha (Yassine et al. 2023).

## Discussion

### Overview of findings

The present review aimed to summarise recent literature on the utility of rsEEG in characterising cognitive dysfunction in PD. Consistent with the general findings of a previous review (Cozac et al. 2016b), studies evaluating spectral characteristics of EEG data consistently reported a widespread shift toward slower oscillatory activity in cases of cognitive decline. Indeed, PD patients with cognitive impairment were observed to have increased delta and theta spectral power, and decreased alpha and beta spectral power, compared to those cognitively unimpaired and healthy participants. Importantly, such neurophysiological abnormalities also correlated with poor performance on assessments of executive functions, visuospatial abilities and global cognition. While the reported topographical distribution of spectral power varied across studies, regional differences generally reflected global patterns, and comparable findings were observed regarding regional power differences and their associations with cognitive function across multiple domains.

PD patients with cognitive impairments were characterised by disruptions in both inter- and intra-network FC, such that connectivity in slower frequency bands (i.e., delta, theta) was generally elevated, while connectivity in faster frequency bands (i.e., alpha, beta) was reduced compared to controls, particularly within frontal and parietal regions. Similar patterns have also been reported in patients without overt cognitive impairment (Conti et al. 2023). By contrast, one study reported increased alpha-band coherence in the intra-hemispheric fronto-parietal regions of PD-MCI. (Carmona Arroyave et al. 2019). Further, one study analysing dynamic FC revealed increased temporal variability in patients with cognitive impairment (Yi et al. 2022). With respect to cognitive abilities, theta connectivity was found to negatively correlate with global cognition as well as with specific cognitive domains including memory, executive functions and visuospatial abilities, while alpha and beta connectivity were generally positively associated with cognitive performance. Notably, one study suggested that the association between connectivity measures and cognitive scores may be influenced by disease duration (Sánchez-Dinorín et al. 2021).

The dynamics of microstate C—considered representative of the salience network (Michel and Koenig 2018)—were altered in all included studies. In particular, the slowing of its dynamics was considered to reflect decreased resting state activity and to be associated with cognitive performance. While only limited alterations were reported in microstates A, B and D across the included studies, the standard four-cluster microstate model may overlook subtler network dynamics. Indeed, one study found no group differences within the canonical A-D microstates, but reported significant effects when applying an eight-cluster solution (Pal et al. 2021).

Variability in the results did not appear to systematically relate to the number of electrodes or measures chosen for analysis.

### Possible underlying mechanisms

A general reduction in higher-frequency spectral power, as well as hypoconnectivity between frontal and parietal regions, may at least partially result from dopamine depletion and reduced D2 receptor availability (Baggio et al. 2015; Biundo et al. 2016; Fiorenzato et al. 2021). In line with this idea, the acute administration of levodopa has been observed to lead to significant increases in alpha and beta spectral power (Melgari et al. 2014) as well as increased connectivity between different resting-state networks (Evangelisti et al. 2019). Interestingly, networks showing the most significant alterations in cognitively impaired PD patients have been observed to correspond to those with the highest energy costs in healthy individuals (Castrillon et al. 2023; Palombit et al. 2022), possibly as a result of higher susceptibility to metabolic changes in the presence of disease (Huang et al. 2007; Yong et al. 2007). Indeed, whereas activity between the fronto-parietal network (FPN) and the DMN is typically strongly anti-correlated in healthy individuals (Fox et al. 2005), aberrant coupling has been documented in PD (Yeager et al. 2024) and may reflect a failure to modulate top-down signals (Biundo et al. 2016). Hyperconnectivity in slow frequencies, on the other hand, may represent an adaptive compensatory response to accumulating pathology (Gorges et al. 2015; Caviness 2014; Wang et al. 2018). In particular, changes in FC patterns may reflect anterior striatal compensation for a more posterior loss of function (Helmich et al. 2010).

Cholinergic degeneration may also contribute to the modulation of cortical oscillations. Previous studies have reported a link between pre-alpha power and atrophy in cholinergic basal forebrain nuclei (Rea et al. 2021). Furthermore, treatment with acetylcholinesterase inhibitors has been shown to partially mitigate the slowing of cortical activity in PD patients experiencing cognitive decline (Bosboom et al. 2009). Of note, several studies reported here found that a decrease in occipital alpha spectral power (Babiloni et al. 2019; Elkholy et al. 2023; Latreille et al. 2016; Liu et al. 2022; Mostile et al. 2019) and connectivity alterations (i.e., increased theta connectivity and decreased alpha connectivity) in occipital regions (Betrouni et al. 2022; Carmona Arroyave et al. 2019; Hassan et al. 2017; Zawiślak-Fornagiel et al. 2023a) were characteristic of PD patients with cognitive impairments. These alterations align with findings from a cholinergic PET study that reported significant reductions in cortical acetylcholinesterase activity primarily in the medial occipital cortex of PDD patients (Shimada et al. 2009). Such changes may be a predictor of faster cognitive decline (Biundo et al. 2016; Kehagia et al. 2012), especially affecting visuospatial functions. Reductions in theta and alpha band connectivity, particularly in prefrontal-limbic and fronto-parietal networks may reflect early cognitive dysfunction driven by the degeneration of cortical and subcortical cholinergic pathways known to play a critical role in both attention and memory processes (Conti et al. 2023). Additionally, hypoconnectivity in alpha networks characterising both body-first and brain-first subtypes of PD further suggests a common early impairment of cholinergic pathways (Conti et al. 2025); the more prominent connectivity disruption in the body-first subtype, with a stronger correlation with cognitive scores, suggests a higher likelihood to progress to a dementia phenotype.

In relation to graph theory metrics, PD-MCI was characterised by reduced efficiency and stability in long-range communication, along with increased temporal variability in alpha band FC, possibly reflecting an early compensatory mechanism that leads to unstable network configurations. In contrast, PDD exhibited a general shift toward hyper-segregation, which may reflect a decline in such compensatory capacities, resulting in rigid and overly segregated networks that are insufficient to support complex cognitive functions (Fiorenzato et al. 2019, 2024).

### Predictive and diagnostic value of qEEG

Consistent with previous findings, EEG-derived spectral power and PLI in the theta (Betrouni et al. 2019; Cai et al. 2021; Chaturvedi et al. 2019; Choi et al. 2024; Cozac et al. 2016a; Kozak et al. 2020; Gschwandtner et al. 2023; Yassine et al. 2023; Zhang et al. 2021) and delta (Babiloni et al. 2019; Betrouni et al. 2019; Chaturvedi et al. 2019; Gschwandtner et al. 2023; Yassine et al. 2023; Zhang et al. 2021) frequency ranges demonstrated the strongest predictive value across multiple models. Additionally, spectral and connectivity measures in the alpha band emerged as promising features (Babiloni et al. 2018a, b, 2019; Betrouni et al. 2019; Choi et al. 2024; Yassine et al. 2023). Both classification into predefined cognitive subgroups and prediction of cognitive trajectories were successfully performed, underscoring the potential of EEG features as biomarkers for PD cognitive decline. Studies employing single-session EEG assessments alongside cognitive measures showed promising results for identification, whereas longitudinal studies incorporating baseline and follow-up data (3–5 years) successfully differentiated patients who remained cognitively stable from those who exhibited decline. Importantly, most predictive models integrated additional variables, including Single Photon Emission Computed Tomography (Arnaldi et al. 2017) and MRI (Zhang et al. 2021) measures, sleep EEG (Latreille et al. 2015) data, motor and non-motor scores (Cozac et al. 2016a; Kozak et al. 2020; Puig-Davi et al. 2024), and plasmatic biomarkers (Liu et al. 2022). While EEG features were not used in isolation, they significantly enhanced the predictive performance of these models, reinforcing their value as a complementary tool in cognitive assessment. A key challenge in implementing such models clinically is the need for sufficiently large datasets, particularly in machine learning approaches, that require accounting for multiple features to ensure generalisability. Although limited availability of extensive subject data remains a barrier in clinical practice, expanding these datasets would not only enhance model robustness but also deepen our understanding of the neurophysiological mechanisms underlying cognitive decline in PD.

### Limitations of available studies

Several factors complicate the interpretation and comparison of the reviewed findings. Choices pertaining to EEG settings, for instance, including the number and placement of electrodes, the reference type, as well as the resting-state protocol (i.e., eyes-open or eyes-closed) varied significantly across studies. Similarly, inconsistencies in patient state during recordings —some conducted in the “ON” state, others in the “OFF” state, and some unspecified—may raise questions about the comparability of EEG results across studies. Efforts to devise a uniform set-up, together with standardised pre-processing and analytical pipelines, might resolve some inconsistencies in the literature and enable a more direct comparison between different studies. In terms of cognitive profiling, a lack of consensus about neuropsychological tests and cut-off values for distinguishing groups of patients (Biundo et al. 2014) may lead to high variability between the nature of cognitive impairments of individuals across studies. Given that differences were reported even between subtypes of MCI patients (Betrouni et al. 2022), standardising methods for cognitive profiling based on domain-specific performance might enhance comparability. Moreover, since higher-order cognitive functions typically rely on coordinated activity across multiple brain regions, studies focusing solely on region-specific—rather than network-based—qEEG measures may fail to capture abnormalities on a larger scale.

### Conclusion

The studies reviewed here reinforce EEG as a promising surrogate marker to support early detection of cognitive abnormalities in PD. Advancements in EEG analysis methods have significantly contributed to the early detection, classification, and monitoring of disease progression, and integrating qEEG with more complex available data—particularly genetic and molecular (Straccia et al. 2022)—may enhance the identification of biologically distinct subtypes. While precision medicine remains constrained by limited large-scale multimodal datasets (Marras et al. 2024), leveraging qEEG-based subtyping may offer a promising strategy to reduce heterogeneity, enable early risk stratification, and inform targeted clinical decision-making as more comprehensive datasets continue to evolve.
